# FATP5 modulates biological activity and lipid metabolism in prostate cancer through the TEAD4-mediated Hippo signaling

**DOI:** 10.3389/fonc.2024.1442911

**Published:** 2024-08-19

**Authors:** Shenyang Liu, Yi He, Zhengqin Gu

**Affiliations:** Department of urology, Xinhua Hospital, Shanghaijiaotong University, Shanghai, China

**Keywords:** FATP5, TEAD4, metabolism, Hippo, YAP1, cancer

## Abstract

**Introduction:**

Prostate cancer (PCa), one of the most prevalent malignant tumors in the genitourinary system, is characterized by distant metastasis and the development of castration-resistant prostate cancer (CRPC), which are major determinants of poor prognosis. Current treatment approaches for PCa primarily involve surgery and endocrine therapy, but effective strategies for managing distant metastasis and CRPC remain limited.

**Methods:**

We utilized qPCR, WB, and other methods to measure the expression levels of respective proteins, concurrently assessing lipid metabolism to validate the role of FATP5 in lipid metabolism. Additionally, we employed bioinformatics analysis and WB techniques to explore the corresponding mechanisms.

**Results:**

In this study, we conducted an analysis of clinical samples and public databases to identify differential expression of FATP5 and further investigated its association with clinical outcomes. Through biochemical and functional experiments, we elucidated the potential underlying mechanisms by which FATP5 facilitates the progression of PCa. Our findings demonstrate that specific upregulation of FATP5 significantly enhances proliferation, migration, and invasion of PCa cell lines, while also modulating lipid metabolism in PCa. Mechanistically, the expression of FATP5 is closely associated with the Hippo signaling pathway, as it promotes the nuclear accumulation of YAP1 by inhibiting AMPK and facilitating the activation of β-catenin and RHOA. Furthermore, the transcription of FATP5 is mediated by TEAD4, and this transcriptional activation requires the involvement of YAP1.

**Discussion:**

FATP5 is highly expressed in prostate cancer and can enhance the biological activity and lipid metabolism of prostate cancer. We have also elucidated that FATP5 is regulated by the Hippo signaling pathway. This provides a new potential target for the treatment of prostate cancer.

## 1 Introduction

PCa is predominantly an adenocarcinoma of epithelial origin, ranking as the second most prevalent malignancy among males worldwide. It accounts for 27% of all incident cases of male cancer (1).There are various treatment options available for localized PCa, with surgical prostatectomy and radiotherapy being the predominant treatment modalities. Despite advancements in clinical interventions for PCa, managing advanced-stage PCa remains a therapeutic challenge. A subset of patients with primary PCa, following initial treatments, will eventually progress to metastatic PCa, which is currently an incurable disease (2). Consequently, there is a crucial need to explore novel biomarkers for the diagnosis and treatment of PCa.

The Hippo pathway is a signaling pathway that plays a critical role in regulating cell growth, proliferation, and organ size. It was initially discovered in fruit flies and has since been found to be highly conserved in mammals, including humans (3). The Hippo pathway consists of a series of protein interactions and phosphorylation events that ultimately control the activity of transcriptional co-activators called Yes-associated protein (YAP) and transcriptional co-activator with PDZ-binding motif (TAZ). When the Hippo pathway is activated, it leads to the phosphorylation and inhibition of YAP/TAZ, preventing their translocation into the nucleus and subsequent activation of target genes involved in cell proliferation and survival. Mutations or dysregulation of the Hippo pathway components have been associated with various diseases, including PCa. Inactivation of the pathway can lead to aberrant YAP/TAZ activity and uncontrolled cell growth, contributing to tumorigenesis and tumor progression (4).

Metabolic reprogramming in the growth of PCa is considered unique in solid tumors because primary prostate tumors tend to enhance oxidative phosphorylation and lipid synthesis, while the elevation of glycolysis is limited (5), this suggests that lipid metabolism may play a more significant role in PCa. Cancer cells acquire fatty acids (FAs) from *de novo* lipid synthesis and exogenous uptake (6). Previous studies on lipid metabolism in PCa have primarily focused on biosynthetic pathways, while research on uptake metabolism has been limited, particularly regarding the FATP (1–6) family. FATP is a member of the solute carrier family 27 (SLC27) and comprises a group of integral membrane proteins with extracellular fatty acid (FA) binding sites, intracellular acyl-CoA synthetase (ACS) active sites, and ATP binding domains (7). *In vitro* and *in vivo* studies have demonstrated that FATP is essential for cancer cell uptake of fatty acids, as well as for growth and invasion (8). And interestingly, in an experiment involving co-culture of PCa cells with adipocytes, it was found that the expression of FATP5 was significantly increased (9). However, the involvement of the six FATP family members in cancer biology remains unclear.

In the present study, we discovered that FATP5 regulates the growth, migration, and invasion of the prostate through the Hippo pathway, while also being deeply involved in lipid metabolism in PCa. Mechanistically, FATP5 promotes the nuclear translocation of YAP1 protein and the transcription of FATP5 is mediated in a YAP1-dependent manner by TEAD4. Additionally, FATP5 is overexpressed in PCa samples, suggesting its potential role in the pathogenesis of PCa.

## 2 Materials and methods

### 2.1 Cell Culture

PCa cell lines, including LNCaP(ATCC CRL-1740, USA),PC-3(ATCC CRL-1435,USA),C4-2B(ATCC CRL-3315,USA),were grown in RPMI-1640 supplemented with 10% fetal bovine serum and 1% penicillin/streptomycin. DU-145(ATCC HTB-81.USA) cells were cultured in DMEM medium supplemented with 10% fetal bovine serum and 1% penicillin/streptomycin. The cells were cultured under the conditions of 37°C, 5% CO2, and passaged at a ratio of 1:2.

### 2.2 Tissue sample

PCa and adjacent tissue samples were collected from patients undergoing urological surgery at Xinhua Hospital affiliated with Shanghai Jiao Tong University School of Medicine. All procedures in this study were approved by the Ethics Committee of Xinhua Hospital affiliated with Shanghai Jiao Tong University School of Medicine, and written informed consent was obtained from each participant.

### 2.3 Tissue microarray

Tissue microarrays (TMAs) were procured from Servicebio Co. Ltd. and immunohistochemical staining was conducted using the FATP5 antibody. Following completion of the staining process, the slides were scanned using a microscope, and the obtained images were analyzed using QuPath software for subsequent assessment of the H score for each tissue core. Integration of the H scores with clinical information was performed to enable comprehensive analysis.

### 2.4 Plasmid construction

The TEAD4 overexpression plasmid, FATP5 overexpression plasmid, pGL3-FATP5(wt), and pGL3-FATP5(mut) plasmids were constructed in-house. Briefly, primers targeting the desired sequences were designed and used for PCR amplification of the target fragments. The amplified fragments were then ligated to the respective plasmids using the In-Fusion^®^ HD Cloning Kit (Takara,Japan) to establish the desired plasmid constructs. The obtained plasmids were subsequently transformed into competent cells for amplification and purification. The pGL3-FATP5(mut) plasmid was generated using the QuickMutation™ Site-directed Gene Mutagenesis Kit (Beyotime,China) through site-directed mutagenesis.

### 2.5 Transfection

pcDNA3.1 Plasmids were transfected using Lipofectamine 3000 (Thermo Fisher,USA) according to the manufacturer’s protocol. Briefly, cells in the logarithmic growth phase were inoculated and cultured overnight in an incubator at 37°C and 5% CO2. The medium was changed to serum-free medium 2 hours before transfection. Then, 5 μg of pcDNA3.1 Plasmids were diluted with 250 μL of serumfree opti-MEM, mixed gently, and kept for 5 minutes. Further, 7.5 μL of Lipofectamine 3000 was diluted using 250 μL of opti-MEM and kept for 5 minutes. Lipofectamine 3000 and plasmids were mixed and kept for 15 minutes. The mixture was added to each Petri dish, and after mixing, cells were cultured in an incubator at 37°C and 5% CO2. 48 hours after culturing, the cells were harvested for transfection efficiency assessment or subsequent experiments.

### 2.6 Establishment of stable expression cell lines

To generate lentivirus, lentivirus vectors (PLVX-shFATP5-Puro, PLVX-shTEAD4-Puro PLVX-shYAP-Puro, 10μg) and lentiviral components (10μg psPAX2 and 2.5μg pMD2.G) were co-transfected into 293T cells in a 10 cm^2^ dish using Polyethylenimine Linear (Yeasen,China). Lentivirus-containing supernatants from 48 and 72 hours post-transfection were collected to infect PCa cells. The stable cell lines were selected using 2μg/ml puromycin for at least 7 days for three passages. Puromycin (1μg/ml) was used to maintain the cells.

### 2.7 Quantitative real-time PCR

Total RNA was extracted using the EZ-press RNA Purification Kit (EZBioscience,China) and concentration was measured by a Nanodrop 2000 instrument. 500 nanogram of total RNA was for reverse transcription using PrimeScript™ RT Master Mix (Takara,Japan). Gene expression measured by qPCR data was collected by QuantStudio 3 Real-Time PCR System with Hieff^®^ qPCR SYBR Green Master Mix (Yeasen,China) and ensure that each experiment is independently replicated three times. Primer sequences are listed in **Supplementary Table S1**.

### 2.8 Western blotting and antibodies

Total proteins were extracted by RIPA lysis buffer (Beyotime,China) together with PMSF (Beyotime,China). The concentration of total protein was determined by BCA protein assay kit (Beyotime,China). After being separated by 10% sodium dodecyl sulfate-polyacrylamide (SDS-PAGE) gels, proteins lysates were transferred to PVDF Membrane (Millipore, USA). The membranes were blocked with 5% BSA for 1hour at room temperature and then immunoblotted at 4°C overnight with primary antibodies: anti-FATP5(1:1000,Proteintech,China), anti-YAP1(1:1000,Proteintech,China), anti-TEAD4(1:1000,Proteintech,China), anti-TAZ(1:1000,Proteintech,China), anti-p-YAP1(1:1000,Proteintech,China),anti-Gapdh(1:1000,Proteintech,China),anti-Tublin(1:1000,Proteintech,China),anti-AMPK(1:1000,Proteintech,China),anti-p-AMPK(1:1000,Proteintech,China),anti-β-catenin(1:1000,Proteintech,China),anti-p-β-catenin(1:1000,Proteintech,China),anti-RHOA(1:1000,Proteintech,China),anti-SMAD2/3(1:1000,Proteintech,China),anti-p-SMAD2/3(1:1000,Proteintech,China),anti-LATS1(1:1000,ABclonal,China),anti-p-LATS1(1:1000,ABclonal,China),anti-N-Cadherin(1:1000,ABclonal,China),anti-E-Cadherin(1:1000,ABclonal,China),anti-Vimentin(1:1000,ABclonal,China),anti-Snail(1:1000,ABclonal,China),anti-YAP1(1:1000,Proteintech,China),anti-Histone H3(Beyotime,China). After incubated with diluted secondary antibodies for 1hour at room temperature, the bands were scanned and analyzed by Tanon 5200 (Tanon, China).

### 2.9 Immunofluorescence

PCa cells were seeded on coverslips. Cells were then washed and treated with 0.3% Triton X-100 for 15 minutes. After blocking with 5% BSA for 1 hour, the YAP1 antibody was added and incubated overnight. The following day, the samples were washed three times with TBST and subsequently incubated with Alexa Fluor 488-labeled Goat Anti-Rabbit IgG (Beyotime,China) at room temperature for 1 hour. After another three washes with TBST, the samples were stained with DAPI(0.1μg/ml). Following a final wash with TBST, the samples were ready for observation under a fluorescence microscope.

### 2.10 EdU incorporation assay

PCa cells were seeded in a 24-well plate and incubated at 37°C for 24 hours. Subsequently, they were treated with 5-ethynyl-20-deoxyuridine (EdU) at a working concentration of 50 μM from the BeyoClick™ EdU Cell Proliferation Kit with Alexa Fluor 488 (Beyotime,China) in 400 μL of culture medium for 2 hours. Following treatment, the cells were washed three times with PBS for 5 minutes each at room temperature. They were then fixed with 4% paraformaldehyde for 30 minutes and incubated with 0.5% Triton X-100 for 15 minutes. Next, nuclear staining was performed using DAPI(0.1μg/ml), followed by thorough rinsing. The samples were observed under a fluorescence microscope, and three random fields were selected for the quantification of positive cells.

### 2.11 CCK8 assay

PCa cells were seeded into 96-well plates at a density of 2000 cells per well with 100 μl medium. 10μl Cell Counting Kit-8 (Beyotime,China) was added to each well at 0 hour, 24 hours, 48 hours, 72 hours and 96 hours after seeding, respectively. For CCK-8 experiments requiring drug treatment, after seeding the cells, allow them to fully adhere to the culture plate. In the experimental group, add different concentrations of the drug, while in the control group, add an equivalent amount of DMSO. Subsequently, follow the same procedure as described earlier. Optical density (OD) at 450 nm was detected by a Multiskan SkyHigh Microplate Spectrophotometer (Thermo Fisher,USA).

### 2.12 Colony formation assay

PCa cells were seeded into 6-well plates at a density of 2000 cells per well. After approximately fifteen days of incubation, the formation of colonies can be observed. Cell colonies were subsequently washed with phosphate buffered saline (PBS), Subsequently, fix the colonies with 10% para-formaldehyde for 30 minutes. Wash the colonies three times with PBS, and then stain them with 0.1% crystal violet for 15 minutes. The representative photographs were taken and the number of colonies were counted.

### 2.13 Transwell assay

PCa cells were resuspended in serum-free media. 5x10^3^ cells in serum-free media were planted into 6.5 mm Transwell^®^ with 8.0 µm Pore Polycarbonate Membrane Insert (Corning, USA) for migration, whereas 5x10^3^cells in serum-free medium were planted into the mentioned chambers, which were precoated with Basement Membrane Matrix High Concentration (Corning, USA) for invasion. After 16 hours of cultivation, fix the chamber in 10% para-formaldehyde and then stain with crystal violet. Subsequently, observe the cells under a microscope and document by capturing images.

### 2.14 BODIPY and Nile red assay for lipid droplet

Regarding lipid droplet staining, we utilized a protocol developed by others as a reference (10). In brief, cells were cultured to a stable state, followed by washing with PBS. Then, a serum-free culture medium was used to dilute BODIPY 493/503 (MCE,USA) to a concentration of 2 μM/L for lipid droplet staining for 30 minutes. Subsequently, the cells were fixed with 4% paraformaldehyde for 30 minutes, followed by DAPI staining for five minutes. After washing with PBS, the stained cells were observed using a fluorescence microscope. Similar to the procedure, Nile Red (MCE,USA) staining was performed by diluting Nile Red to a concentration of 1 μM/L for 30 minutes. Fluorescence intensity was observed and recorded under a fluorescence microscope. All samples from the same experiment were imaged by using the same settings (gain, laser power). For lipid uptake, a serum-free culture medium was used to dilute BODIPY 500/510 C1, C12 (MCE,USA) to a concentration of 6 μM/L. The cells were then incubated at 37°C for 30 minutes. Subsequently, fluorescence intensity was measured using a fluorescence microscope. Additionally, stained cells were analyzed using a flow cytometer (BD,USA), and the data were analyzed using FlowJo software.

### 2.15 Dual-Luciferase reporter gene assay

The experiment was performed as per the standard protocol. Briefly, the cells were transfected with Lipofectamine 3000 (Thermo Fisher,USA), and then Dual Luciferase Reporter Gene Assay Kit (Yeasen,China) was used. After cell lysis and thorough mixing, transfer the lysate into a 96-well plate for further testing. The fluorescence analyzer was used to detect firefly and renilla luciferin enzyme activity. Then the ratio of firefly to renilla luciferase was calculated.

### 2.16 *In vivo* animal studies

A subcutaneous xenograft model was employed to investigate the effects of oeFATP5, shTEAD4, and TED-347 on tumor growth. A total of six 4-5-week-old male nude mice (SLAC, China) were randomly divided into the experimental and control groups. One million PC-3 cells that overexpressing FATP5 or shTEAD4 were mixed with a 1:1 ratio of serum-free medium and matrix gel and injected into the axillary region of the nude mice. In the case of the TED-347 experimental group, mice were injected with TED-347 at a dose of 20 mg/kg for three times on days 1, 7, and 14, while the control group received an equivalent volume of DMSO. Tumor size was measured every three days, and on day 21, the mice were euthanized to measure tumor size and weight. All animal experiments were conducted in accordance with the guidelines of the Ethics Committee of Xinhua Hospital affiliated with Shanghai Jiao Tong University School of Medicine.

### 2.17 Drug treatments

All drugs, including TED-347 (MCE), Enzalutamide (MCE,USA) and Leptomycin B (Beyotime,China) were dissolved in DMSO to prepare a 10 mM stock solution. For *in vitro* assays, the stock solutions were diluted with serum-free culture medium to the desired concentrations and added to the cell culture medium. The cells were treated for different durations according to experimental requirements. The control group received the corresponding amount of DMSO without any drug treatment.

### 2.18 IC50 assay

PCa cells were seeded into 96-well plates at a density of 5000 cells per well with 100 μl medium. After the cells have fully adhered, different concentrations of Enzalutamide are sequentially added to the experimental groups, while the control group receives an equal volume of DMSO. The cells are then incubated undisturbed for 48 hours. Following the incubation period, 10 μL of CCK-8 reagent is added to each well, and the cells are further incubated for 90 minutes. Subsequently, the absorbance at 450 nm is measured using Spectrophotometer. The data is analyzed using GraphPad Prism 9.0 software to calculate the IC50 value for each group of cells.

### 2.19 Malondialdehyde levels

The content of malondialdehyde (MDA) can be determined by measuring thiobarbituric acid (TBA) using Lipid Peroxidation MDA Assay Kit (beyotime, China). The experiments were performed as per the kit’s manual. In simple terms, the procedure involves collecting cells and determining their protein concentration using the BCA assay. The working solution is then added to the cell lysates or standard samples. The mixture is heated at a minimum of 100°C in a metal bath for at least 15 minutes. After centrifugation, the supernatant is collected, and the optical density (OD) is measured at 532 nm. The MDA concentration is calculated by comparing the obtained OD values with a standard concentration curve.

### 2.20 ROS levels

The experiment is conducted following the instructions provided in the Reactive Oxygen Species Assay Kit (Beyotime,China). In brief, after cell adherence, 1 ml of diluted DCFH is added to the culture dish. The dish is then incubated at 37°C for 20 minutes. Subsequently, the cells are washed three times with serum-free culture medium. The fluorescence microscope is set to an excitation wavelength of 488 nm and an emission wavelength of 525 nm. Under consistent imaging parameters, photographs are taken for both the experimental and control groups. Subsequently, the average fluorescence intensity is calculated for each group, allowing for the determination of the reactive oxygen species (ROS) levels.

### 2.21 NADPH levels

The experiment is conducted following the instructions provided in the NADP^+^/NADPH Assay Kit with WST-8.After cell collection, the cells are lysed using NADP+/NADPH extraction buffer. The lysate is divided into two portions. One portion is centrifuged at 12,000 g for 10 minutes at 4°C, and the supernatant is collected. A 200 μl sample is taken for further analysis. The other portion of the sample is subjected to a 60°C water bath treatment for 30 minutes, followed by centrifugation at 10,000 g for 5 minutes at 4°C. The supernatant is collected and mixed with G6PDH working solution and a color reagent. The mixture is then incubated at 37°C in the dark for 20 minutes. The absorbance of the sample at 450 nm is measured using the Multiskan SkyHigh Microplate Spectrophotometer. The NADPH oxidase activity is calculated based on the standard curve.

### 2.22 Bioinformatics analysis

We utilized the GEPIA2 web server to analyze the expression profile of the FATP protein family in the TCGA dataset. The QuPath software was employed for the analysis of tissue microarray staining. The H-score was calculated using the formula H-score=Σ(Pi*i), where Pi represents the proportion of positive cells and i represents the staining intensity. Based on the expression status of FATP5 in prostate cancer patients from the TCGA database, they were divided into the FATP5 high-expression group and the FATP5 low-expression group. Gene Set Enrichment Analysis (GSEA) V4.3.3 and MSigDB were used for gene enrichment analysis.

### 2.23 Statistical analysis

The data are presented as mean ± standard deviation (SD). GraphPad Prism 9.0 software is used for data analysis. Statistical analysis includes unpaired two-tailed t test, paired t test, two-way analysis of variance (ANOVA) followed by Tukey’s multiple comparisons test. A p-value less than 0.05 (P < 0.05) is considered statistically significant.

## 3 Results

### 3.1 FATP5 shows upregulated expression in PCa tissues and cell lines

**Figure 1:**
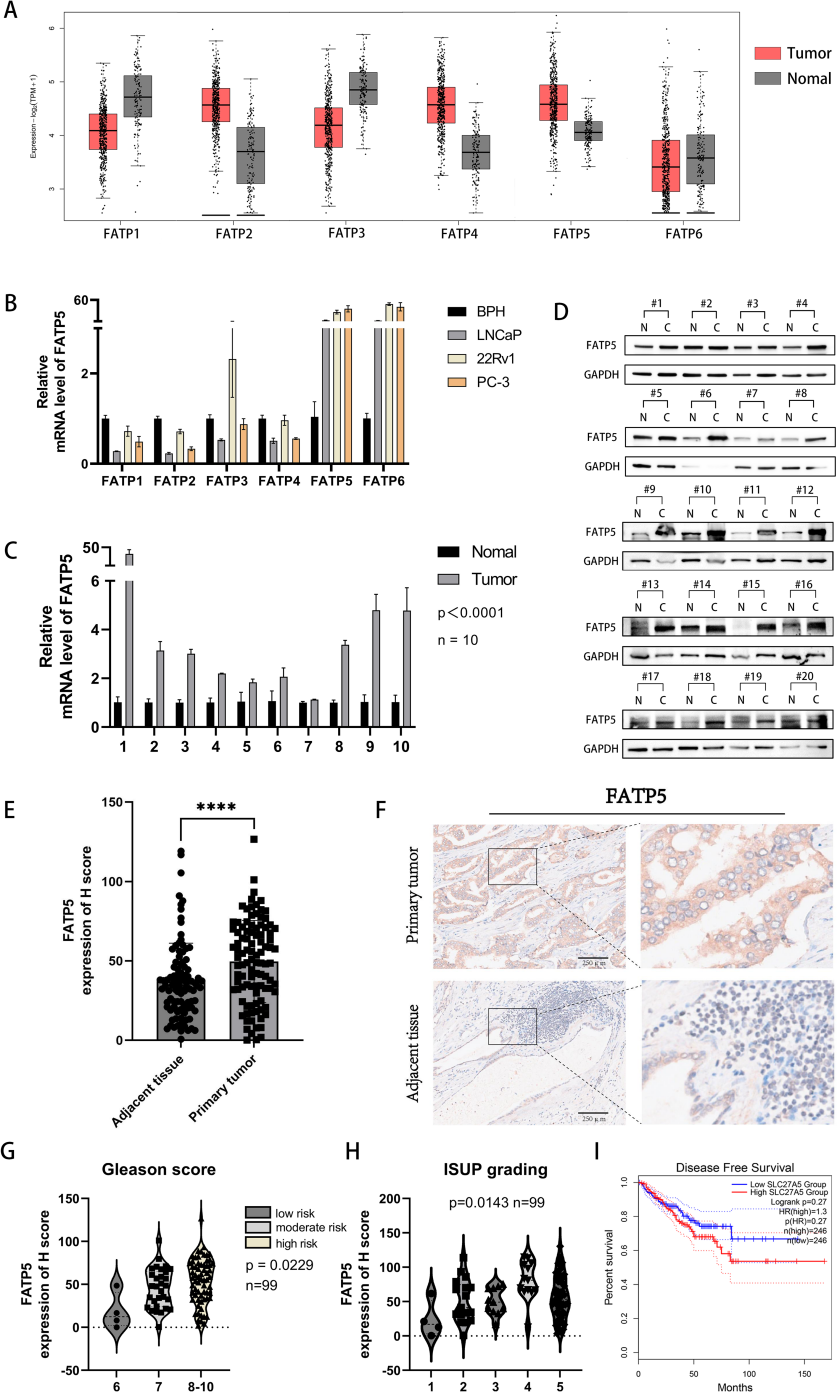
FATP5 shows upregulated expression in PCa tissues and cell lines. **(A)** Download the expression transcriptome profile of FATPs mRNA in PCa tissues (n = 492) and adjacent normal tissues (n = 192) from the TCGA-PRAD database. **(B)** Assess the mRNA expression levels of FATPs in PCa cell lines. **(C)** Determine the expression levels of FATP5 mRNA in cancer and adjacent tissues in 10 PCa patients. **(D)** Measure the protein expression levels of FATP5 in cancer and adjacent tissues in 20 PCa patients. **(E)** Generate H score scoring charts based on FATP5 immunostaining in 100 patient tissue chips. **(F)** Present immunohistochemistry staining results for FATP5. **(G, H)** Investigate the correlation between FATP5 and Gleason score and ISUP grading based on clinical data from patient tissue chips. **(I)** FATP5 and disease-free survival of prostate cancer. Mean ± SD, n = 3. ****p < 0.0001.

To investigate the role of the FATP protein family in the occurrence and progression of PCa, we initially analyzed the expression profiles of the FATP family in PCa using publicly available TCGA data. We found that FATP (2, 4, 5) expression was upregulated, while FATP (1, 3, 6) expression was downregulated in PCa (**Figure 1A**). Furthermore, we examined the mRNA expression of the FATP family in several common PCa cell lines (LNCaP, PC-3, DU-145) compared to benign prostatic hyperplasia (BPH) cell lines. In contrast to the TCGA database, only FATP (5, 6) showed increased expression in all three cell lines (**Figure 1B**). Similar results were observed in two additional PCa cell lines, DU-145 and C4-2B (**Supplementary Figure S1A**). Consequently, we hypothesized that FATP5 may play a crucial role in the development of PCa. Subsequently, we obtained paired tissue samples from Xinhua Hospital affiliated with Shanghai Jiao Tong University School of Medicine, to confirm the expression levels of FATP5 in PCa tissues. Ten pairs of samples were used for qRT-PCR analysis, and twenty pairs were used for Western blotting. The results showed that FATP5 expression was higher in PCa tissues compared to adjacent normal tissues (**Figures 1C, D**). Tissue microarray (TMA), a technique used for high-throughput analysis of tissue samples, was employed. The TMA used in our study included tissue samples from one hundred PCa patients. Quantitative analysis of the staining results for each tissue spot was performed using QuPath software, and the corresponding H score was obtained. The results demonstrated significantly higher expression levels of FATP5 in tumor tissues compared to normal adjacent tissues (**Figure 1E**). Additionally, immunohistochemistry staining confirmed significantly stronger staining for FATP5 in cancer tissues compared to adjacent non-cancerous tissues (**Figure 1F**). Subsequently, based on the Gleason score, the samples were divided into three groups: low-risk group with a Gleason score of 6, intermediate-risk group with a Gleason score of 7, and high-risk group with a Gleason score of 8-10. It was observed that as the risk level increased, the expression of FATP5 also significantly increased (**Figure 1G**). According to the ISUP grading results, there is a gradual increase in the expression of FATP5 with higher grades. This indicates that in PCa, as the malignancy level increases, there is a corresponding elevation in the expression level of FATP5. (**Figure 1H**). Furthermore, FATP5 also showed a trend associated with DFS (**Figure 1I**). Furthermore, FATP5 also showed a trend associated with DFS (**Figure 1I**).Overall, these findings confirm the high expression of FATP5 in patient tissues and cell lines, as well as its association with Gleason score risk stratification and ISUP grading.

### 3.2 FATP5 is involved in regulating the proliferation and migration of PCa cells

**Figure 2:**
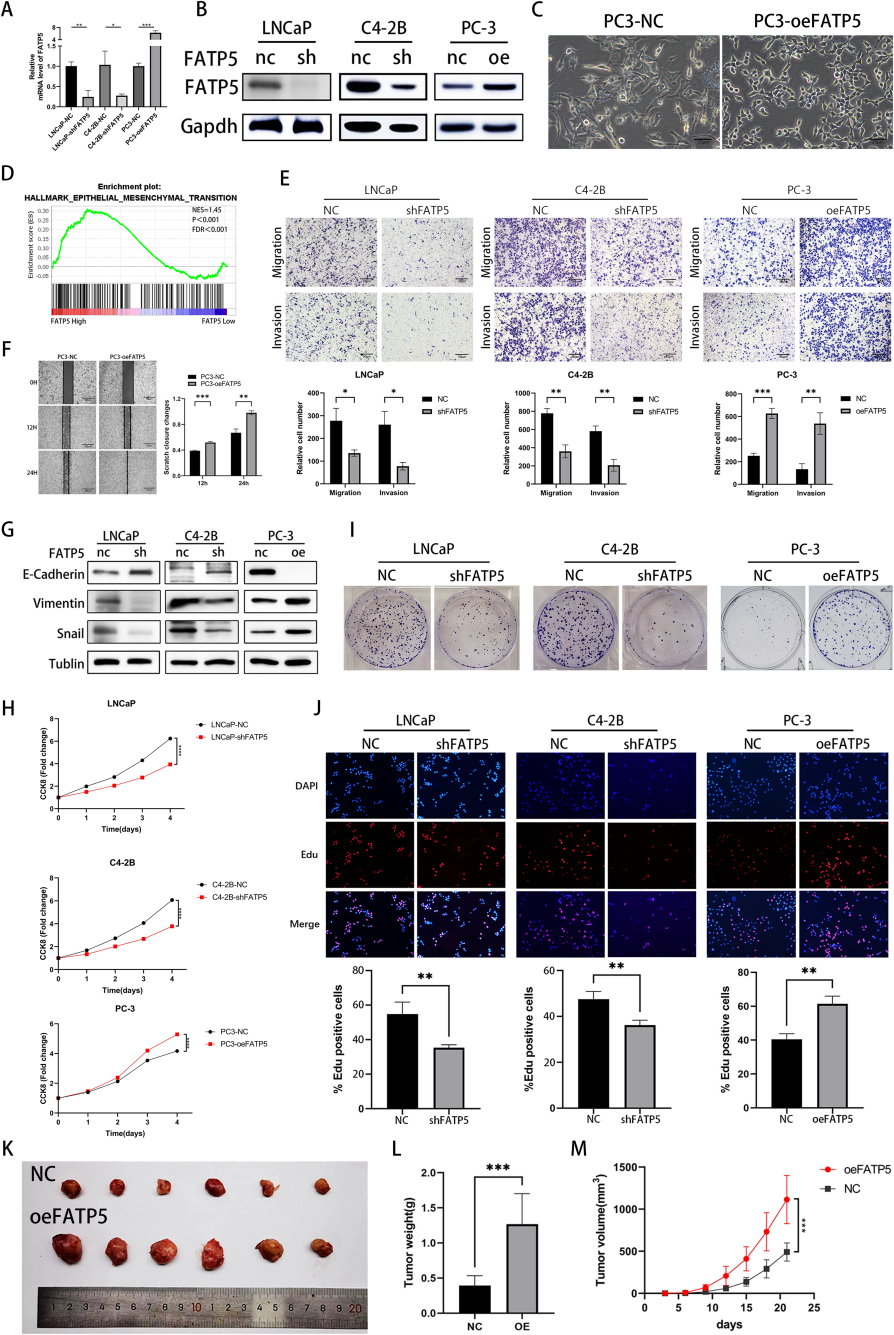
FATP5 is involved in regulating the proliferation and migration of PCa cells **(A, B)** Evaluate the protein and mRNA levels of FATP5 after overexpression or knockdown. **(C)** Morphologically observe PC-3 cells after overexpressing FATP5 under light microscopy. **(D)** Perform Gene Set Enrichment Analysis (GSEA) to assess the correlation between EMT signaling pathways and FATP5 mRNA levels based on TCGA database. **(E)** Investigate the migration and proliferation abilities of PCa cells using the Transwell assay (Magnification: 100×). **(F)** Validate the planar migration capability of PCa cells using the scratch assay. **(G)** Assess the expression levels of EMT-related markers after FATP5 knockdown or overexpression. **(H)** Determine cell growth curves using the CCK8 assay. **(I)** Conduct colony formation assays to observe the size and quantity of PCa cell colonies. **(J)** Assess proliferation ability of PCa cells using the EdU assay. **(K–M)** Measure tumor size, weight, and volume in *in vivo* experiments. Mean ± SD, n = 3. *p < 0.5, **p < 0.01, ***p < 0.001, and ****p < 0.0001.

To investigate the impact of FATP5 on the proliferation and metastasis of PCa, extensive functional studies were conducted using PCa cell lines. Lentiviral vectors were used to overexpress FATP5 and introduce short hairpin RNA (shRNA) targeting FATP5 into the relevant cell lines, resulting in stable cell lines with low (LNCaP-shFATP5, C4-2B-shFATP5) and high (PC3-oeFATP5) expression of FATP5 (**Figures 2A, B**). Metastasis is a major cause of mortality in PCa (11). After overexpressing FATP5, we observed significant changes in the morphology of PC-3 cells (**Figure 2C**). Subsequently, gene set enrichment analysis (GSEA) based on TCGA database was employed to investigate the biological processes regulated by FATP5 and its impact on PCa carcinogenesis and progression (**Figure 2D**). The findings indicated a significant association between FATP5 and EMT signaling characteristics, suggesting a potential influence of FATP5 on EMT processes. Transwell assays were performed in the aforementioned cell lines to determine the role of FATP5 in cell migration. As expected, LNCaP-shFATP5 and C4-2B-shFATP5 cells exhibited decreased migration capability, while PC-3-oeFATP5 cells showed increased migration ability (**Figure 2E**). Similarly, transwell assays were employed to assess cell invasion, and consistent with previous observations, LNCaP-shFATP5 and C4-2B-shFATP5 cells demonstrated reduced invasive potential, whereas PC3-oeFATP5 cells exhibited enhanced invasion (**Figure 2E**). Scratch assays were also conducted, revealing increased migration ability upon FATP5 overexpression (**Figure 2F**). Furthermore, multiple EMT markers were examined in the aforementioned cell lines to validate the results. Western blot analysis demonstrated decreased expression levels of Vimentin and Snail, while E-cadherin expression increased in LNCaP-shFATP5 and C4-2B-shFATP5 cells, with opposite results observed in FATP5 overexpressing cell lines (**Figure 2G**). In summary, FATP5 overexpression promoted PCa EMT *in vitro*, while knockdown of FATP5 inhibited PCa EMT.

We also assessed the influence of FATP5 on the proliferation of PCa cells. The CCK-8 assay was employed to evaluate the cellular proliferation capacity of PCa cells. The results revealed a significant reduction in the proliferation rate of cells lacking FATP5 (LNCaP-shFATP5, C4-2B-shFATP5) (**Figure 2H**). Conversely, overexpression of FATP5 resulted in an elevated proliferation rate of PCa cells (**Figure 2H**). Clonogenic assays demonstrated that FATP5 knockdown led to smaller and fewer colony formations in PCa cells, while FATP5 overexpression yielded the opposite results (**Figure 2I**, **Supplementary Figure S1B**). In addition, we conducted EdU assays, where both fast-proliferating and slow-proliferating cells incorporate EdU during active DNA synthesis, and the signal intensity of EdU in cells indicates the proliferation rate. Our results showed that the proportion of EdU-positive cells was lower in FATP5 knockdown cells, while it was higher in FATP5 overexpressing cells. (**Figure 2J**). Given that our functional studies on FATP5 were conducted *in vitro*, further *in vivo* functional validation was performed. PC-3 cells overexpressing FATP5 were implanted subcutaneously in nude mice, and tumor size was measured every 3 days. On the 21st day, the subcutaneous tumors were excised. The results demonstrated that overexpression of FATP5 significantly increased tumor weight and volume (**Figures 2K–M**). In summary, these *in vitro* and *in vivo* functional investigations clearly indicate that FATP5 plays a promoting role in the migration, invasion, and proliferation of tumors.

### 3.3 FATP5 significantly promotes lipid accumulation in PCa cells

**Figure 3:**
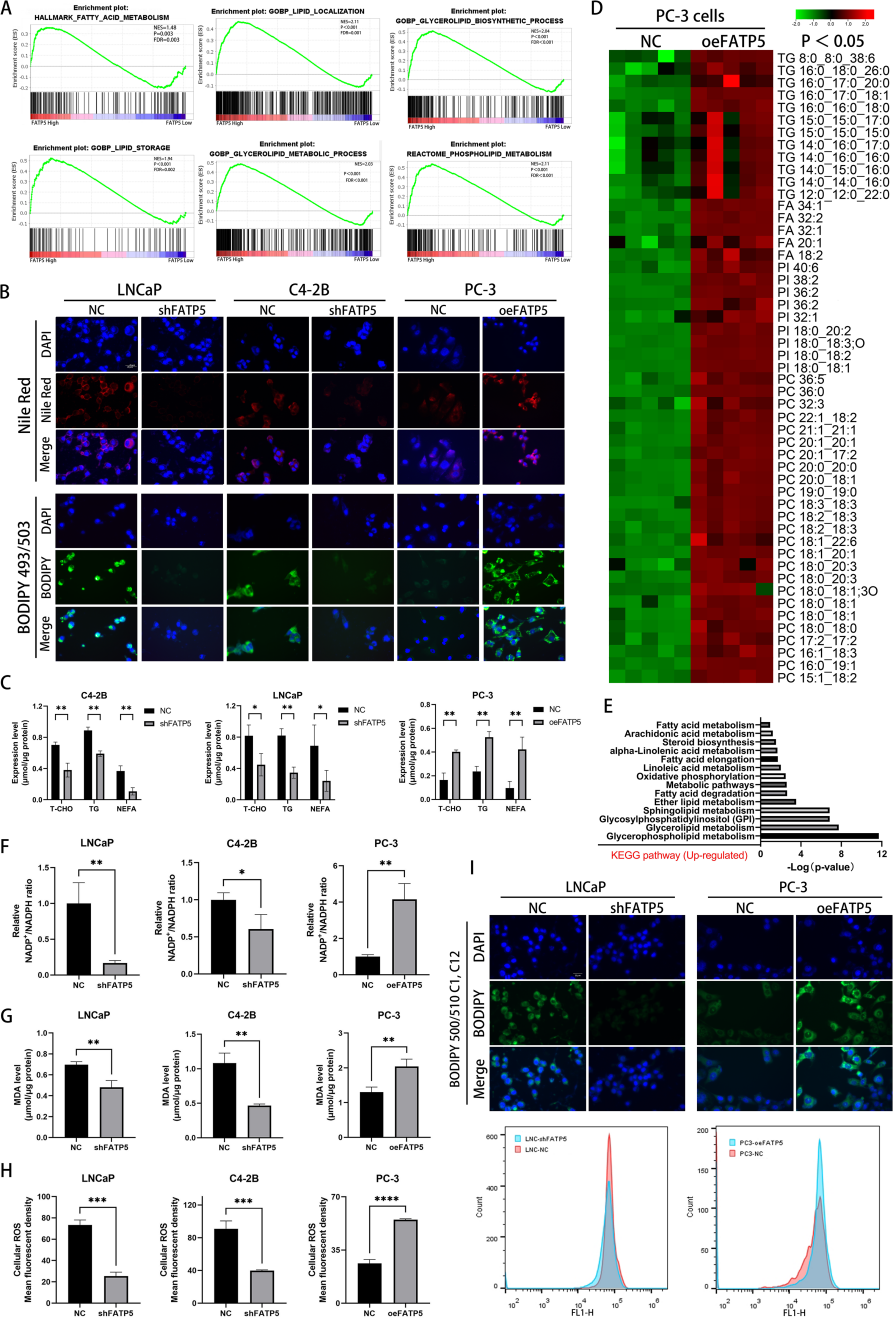
FATP5 significantly promotes lipid accumulation in PCa cells. **(A)** Perform GSEA to explore the correlation between lipid metabolism-related pathways and FATP5 mRNA levels based on TCGA database. **(B)** Stain FATP5-overexpressing or knockdown cell lines with Nile red and BODIPY 493/503 (Magnification: 400×). **(C)** Quantify the content of corresponding lipids in FATP5-overexpressing or knockdown cell lines. **(D)** Employ LC/MS lipidomics to detect intracellular lipids in stable or unstable FATP5-overexpressing PC-3 cells (n = 5), highlighting statistically significant metabolites (p < 0.05) in the heatmap using red and green colors. **(E)** Present KEGG pathway enrichment analysis of metabolite enrichment based on LC/MS lipidomics analysis. **(F–H)** Analyze the NADP+/NADPH ratio, MDA levels, and ROS levels in cells after FATP5 overexpression or knockdown, normalized to total protein content. **(I)** Analyze fluorescence intensity of BODIPY 500/510 C1,C12 staining using flow cytometry. Mean ± SD, n = 3. *p < 0.5, **p < 0.01, ***p < 0.001, and ****p < 0.0001.

In PCa cells, enhanced lipid accumulation is often observed (12). Next, we performed gene set enrichment analysis (GSEA) to analyze the enrichment of lipid metabolism-related genes in the TCGA database after FATP5 alteration. The results showed a strong correlation between pathways related to lipid metabolism and high expression of FATP5 (**Figure 3A**), suggesting that FATP5 is involved in the regulation of lipid metabolism in PCa. To validate this hypothesis, we stained cells with Nile red and BODIPY 493/503 in three cell lines overexpressing or knockdown of FATP5. The results revealed that cells with high FATP5 expression exhibited increased lipid accumulation, while cells with low FATP5 expression showed decreased lipid content (**Figure 3B**). Subsequently, we measured the levels of triglycerides (TG), total cholesterol (T-CHO), and non-esterified fatty acids (NEFA) as quantitative indicators of lipid accumulation in the three FATP5-treated cell lines. As shown in **Figure 3C**, cells with high FATP5 expression had elevated levels of TG, T-CHO, and NEFA, while the opposite was observed in cells with low FATP5 expression (**Figure 3C**).

Subsequently, we conducted liquid chromatography/mass spectrometry (LC/MS) lipidomic analysis on PC-3 cells with either overexpression of FATP5. The results, depicted in **Figure 3D**, demonstrated a significant increase in the levels of triglycerides (TG), fatty acids, phosphatidylcholine (PC), and phosphatidylinositol (PI) in PC-3 cells with stable FATP5 overexpression compared to the control cells. Furthermore, KEGG metabolic pathway analysis revealed the correlation of differential metabolites with multiple lipid metabolism pathways (**Figure 3E**).

Considering the anticipated association between lipid accumulation and increased reactive oxygen species (ROS) generation, we quantified the NADP^+^/NADPH ratio, intracellular ROS levels, and lipid peroxidation levels in PCa cell lines. As expected, FATP5 overexpression resulted in an elevation of the NADP^+^/NADPH ratio within the cells (**Figure 3F**), an induction of lipid peroxidation, including elevated malondialdehyde (MDA) levels (**Figure 3G**) and an augmentation of intracellular ROS levels (**Figure 3H**). Conversely, downregulation of FATP5 exerted opposing effects on the NADP^+^/NADPH ratio, intracellular ROS levels, and MDA levels in PCa cells (**Figures 3F–H**).

In order to elucidate the reasons for lipid accumulation, we supplemented the culture medium with BODIPY 500/510 C1, C12 fluorescent probe and performed flow cytometry analysis to measure the fluorescence intensity of PCa cells with different FATP5 treatments after thirty minutes of incubation. It was observed that following FATP5 overexpression, the fluorescence intensity was significantly enhanced compared to the control group, while cells with FATP5 knockdown exhibited lower fluorescence intensity (**Figure 3I**). These experimental results suggest that the lipid accumulation induced by FATP5 is at least partially attributed to enhanced uptake of exogenous fatty acids. Based on these findings, we propose that FATP5 enhances lipid accumulation in PCa and that part of this accumulation is due to increased uptake of exogenous fatty acids. Additionally, FATP5 also promotes ROS generation and lipid peroxidation levels.

### 3.4 FATP5 is regulated by the Hippo signaling pathway and promotes the nuclear translocation of YAP1 protein

**Figure 4:**
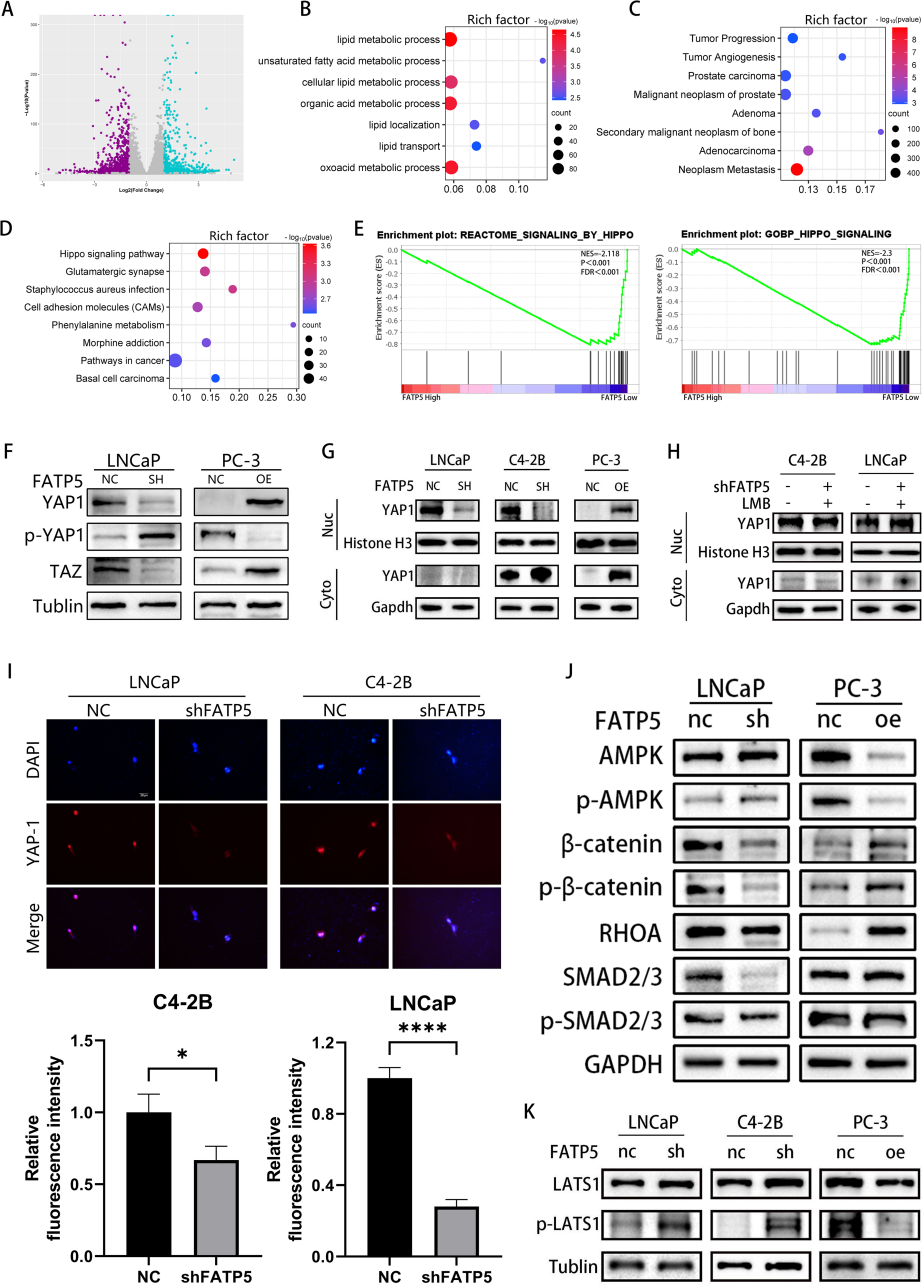
FATP5 is regulated by the Hippo signaling pathway and promotes the nuclear translocation of YAP1 protein. **(A)** Generate volcano plots to display the differential gene expression profile following FATP5 knockdown. **(B–D)** Conduct KEGG pathway analysis on the differentially expressed genes identified from RNA-seq data. Criteria for selection: |Log2(fold change)| ≥ 2 and p-value ≤ 0.05. **(E)** Investigate the correlation between the Hippo signaling pathway and FATP5 mRNA levels using Gene Set Enrichment Analysis (GSEA) based on data from the TCGA database. **(F)** The expression status of key proteins in the Hippo signaling pathway following knockdown or overexpression of FATP5. **(G)** Measure the expression levels of YAP1 in the nuclear and cytoplasmic fractions following FATP5 overexpression or knockdown. Cyto, cytoplasm; nuc, nucleus. **(H)** Determine the expression levels of YAP1 in the nuclear and cytoplasmic fractions after the addition of Leptomycin B (LMB) to the cells. **(I)** Perform immunofluorescence staining to examine the fluorescence intensity of nuclear YAP1 (Magnification: 400×). **(J, K)** The expression profile of key proteins in the signaling pathway following knockdown or overexpression of FATP5. Mean ± SD, n = 3. *p < 0.5, ****p < 0.0001.

C4-2B cells with FATP5 knockdown were selected for whole transcriptome sequencing to gain deeper insights into the mechanistic role of FATP5. The transcriptomic analysis demonstrated significant alterations in gene expression, with 1,727 genes being downregulated and 1,064 genes being upregulated (**Figure 4A**). Subsequent KEGG pathway analysis of the differentially expressed genes revealed enrichment in multiple lipid metabolism pathways and tumor metastasis pathways upon FATP5 knockdown (**Figures 4B, C**), the reliability of the previous experimental results is verified again. Furthermore, a notable enrichment of differentially expressed genes was observed in the Hippo pathway (**Figure 4D**), which was further validated by Gene Set Enrichment Analysis (GSEA) (**Figure 4E**). Therefore, we reasonably suspect that FATP5 is regulated by the Hippo signaling pathway.

We subsequently validated this in cells by examining the levels of total YAP1, TAZ, and phosphorylated YAP1 (p-YAP1) in FATP5 knockdown or overexpression cell lines. The results showed that upon FATP5 knockdown, the levels of total YAP1 and TAZ decreased, while the level of phosphorylated YAP1 increased. Conversely, cells overexpressing FATP5 exhibited the opposite results (**Figure 4F**). As YAP1 protein requires nuclear localization to exert its function (13), we further investigated the impact of FATP5 on YAP1 nuclear translocation. We isolated nuclear and cytoplasmic proteins from PCa cells and measured the expression levels of YAP1 in each fraction (**Figure 4G**). Following FATP5 knockdown, the level of YAP1 in the nucleus significantly decreased, while overexpression of FATP5 led to a notable increase in nuclear YAP1 protein. We also employed Leptomycin B (LMB), a nuclear export inhibitor that prevents protein transport from the nucleus to the cytoplasm (14). Concurrently with FATP5 knockdown, we added LMB to the culture medium and subsequently assessed the nuclear YAP1 content. The results showed that the addition of LMB partially attenuated the reduction in nuclear YAP1 (**Figure 4H**). Furthermore, we performed immunofluorescence staining on treated cells and calculated the average fluorescence intensity in the nuclear region. The results revealed a significant reduction in nuclear fluorescence intensity following FATP5 knockdown (**Figure 4I**).

To investigate the mechanism by which FATP5 promotes YAP1 nuclear translocation, we examined the expression levels of intracellular ACSL4 (**Supplementary Figure S2A**). Overexpression of FATP5 enhanced the levels of intracellular fatty acids, while ACSL4, as a member of the ACSL protein family, facilitates the binding of long-chain fatty acids to coenzyme A, forming acyl-CoA. Acyl-CoA is a key intermediate in fatty acid β-oxidation. Consequently, we subsequently assessed the expression of key genes involved in fatty acid β-oxidation and found that FATP5 was able to promote the expression of these key genes (**Supplementary Figure S2B**). The enhanced fatty acid β-oxidation inevitably leads to an increase in intracellular ATP levels, consistent with our expectations, as FATP5 elevated the intracellular ATP content (**Supplementary Figure S2C**). The elevated ATP inhibited the activation of the AMPK signaling pathway, which has been demonstrated to promote the degradation of YAP1 (15). In summary, we propose that FATP5 enhances intracellular ATP levels by augmenting fatty acid β-oxidation, thereby inhibiting the activation of the AMPK signaling pathway and facilitating YAP1 nuclear translocation. We also examined the expression of key proteins involved in multiple signaling pathways regulating YAP1 nuclear translocation, including the HGF-induced β-catenin pathway, TGF-β-induced SMAD pathway, and Ephrin A2-induced RHO-dependent pathway. The results showed (**Figure 4J**) that after FATP5 overexpression, the protein expression of β-catenin and phosphorylated β-catenin at S552 (p-β-catenin) significantly increased, as did the protein level of RHOA. However, the expression levels of SMAD2/3 and phosphorylated SMAD2/3 did not show significant changes. In cells with FATP5 knockdown, the opposite protein expression patterns were observed. Additionally, we examined the levels of Large Tumor Suppressor Kinase 1 (LATS1) and phosphorylated LATS1 (p-LATS1). Phosphorylation of LATS1 in the Hippo pathway leads to increased degradation of YAP1 and inhibition of downstream gene transcription (16). The results revealed that in PCa cells with FATP5 overexpression, the level of phosphorylated LATS1 significantly decreased, whereas in cells with FATP5 knockdown, the phosphorylation level of LATS1 significantly increased (**Figure 4K**). Previous studies have demonstrated that the AMPK signaling pathway can directly regulate the activity of β-catenin and RHOA, and activation of AMPK inhibits the activity of β-catenin and RHOA (17, 18). Therefore, our findings suggest that knockdown of FATP5 reduces lipid metabolism in PCa and activates the AMPK signaling pathway, thereby inhibiting the activity of β-catenin and RHOA, suppressing YAP1 nuclear translocation, leading to YAP1 accumulation in the cytoplasm, phosphorylation, and subsequent degradation, and ultimately inhibiting downstream gene transcription.

### 3.5 TEAD4 positively regulates the transcription of FATP5 and influences the activity of PCa

**Figure 5:**
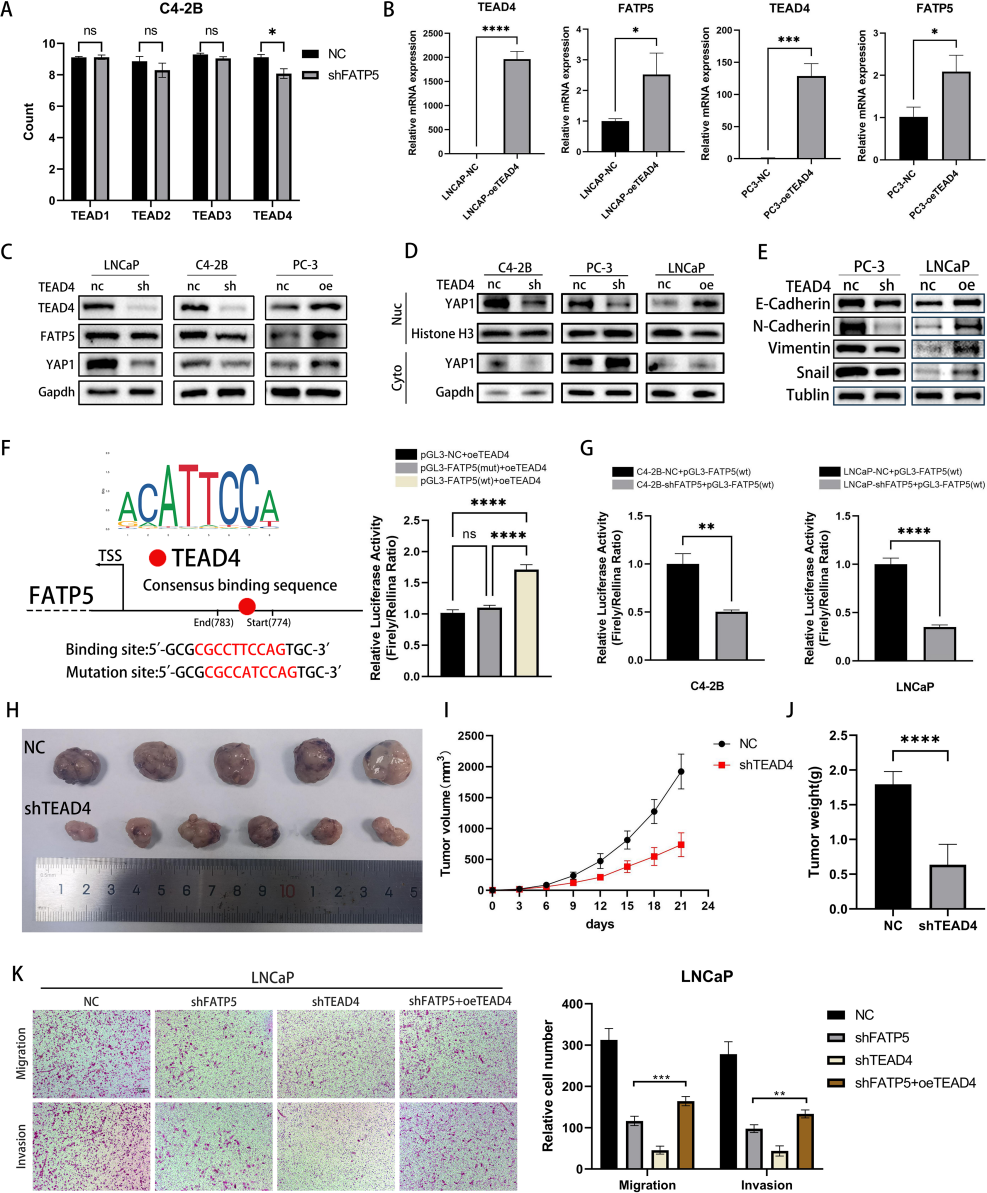
TEAD4 positively regulates the transcription of FATP5 and influences the activity of PCa. **(A)** Quantify the read counts of TEAD family members in the RNA-seq data. **(B, C)** Determine the mRNA and protein levels of TEAD4 and FATP5 in PCa cells following transfection with TEAD4 overexpression. **(D, E)** Assess the protein levels of YAP1 in the nuclear and cytoplasmic fractions, as well as the protein levels of corresponding EMT markers, after TEAD4 knockdown or overexpression. **(F, G)** Present the results of the dual-luciferase reporter gene assay. TSS, Transcription Start Site; wt, wild type; mut, mutant. **(H–J)** Evaluate the tumor size, weight, and volume in mice following TEAD4 knockdown. **(K)** Conduct migration and invasion experiments on specific PCa cells (Magnification: 100×). Mean ± SD, n = 3. *p < 0.5, **p < 0.01, ***p < 0.001, and ****p < 0.0001. ns, no significance.

Upon translocation into the nucleus, YAP1 protein usually forms a complex with the TEAD family to exert its functional role (19). TEAD carries a TEA DNA-binding domain near its N-terminus and a YBD (YAP binding domain) near its C-terminus (20). Based on our transcriptomic data, we observed a significant downregulation of TEAD4 expression following FATP5 knockdown (**Figure 5A**). Therefore, we had a rationale to suspect that FATP5 transcription might be regulated by TEAD4. To validate our hypothesis, we overexpressed TEAD4 in PCa cells and measured the mRNA expression of FATP5. As shown in **Figure 5B**, FATP5 expression was significantly increased upon TEAD4 overexpression, which was further confirmed by Western blot analysis (**Figure 5C**). Similarly, the reduction of TEAD4 also affected the nuclear accumulation of YAP1 (**Figure 5D**). Correspondingly, there were alterations in EMT markers (**Figure 5E**). Luciferase reporter gene analysis demonstrated that upregulation of TEAD4 enhanced the luciferase activity of the FATP5 promoter (**Figure 5F**). Subsequently, using JASPAR, we predicted the TEAD4 binding sites in the FATP5 promoter region and performed site-directed mutagenesis using a mutation kit to disrupt these binding sites. The luciferase activity was then measured, revealing a decrease in luciferase activity upon mutation (**Figure 5F**). Furthermore, we conducted experiments in two additional FATP5 knockdown cell lines and observed a significant decrease in luciferase activity (**Figure 5G**). In conclusion, we can infer that TEAD4 mediates the transcription of FATP5.

**Figure 6:**
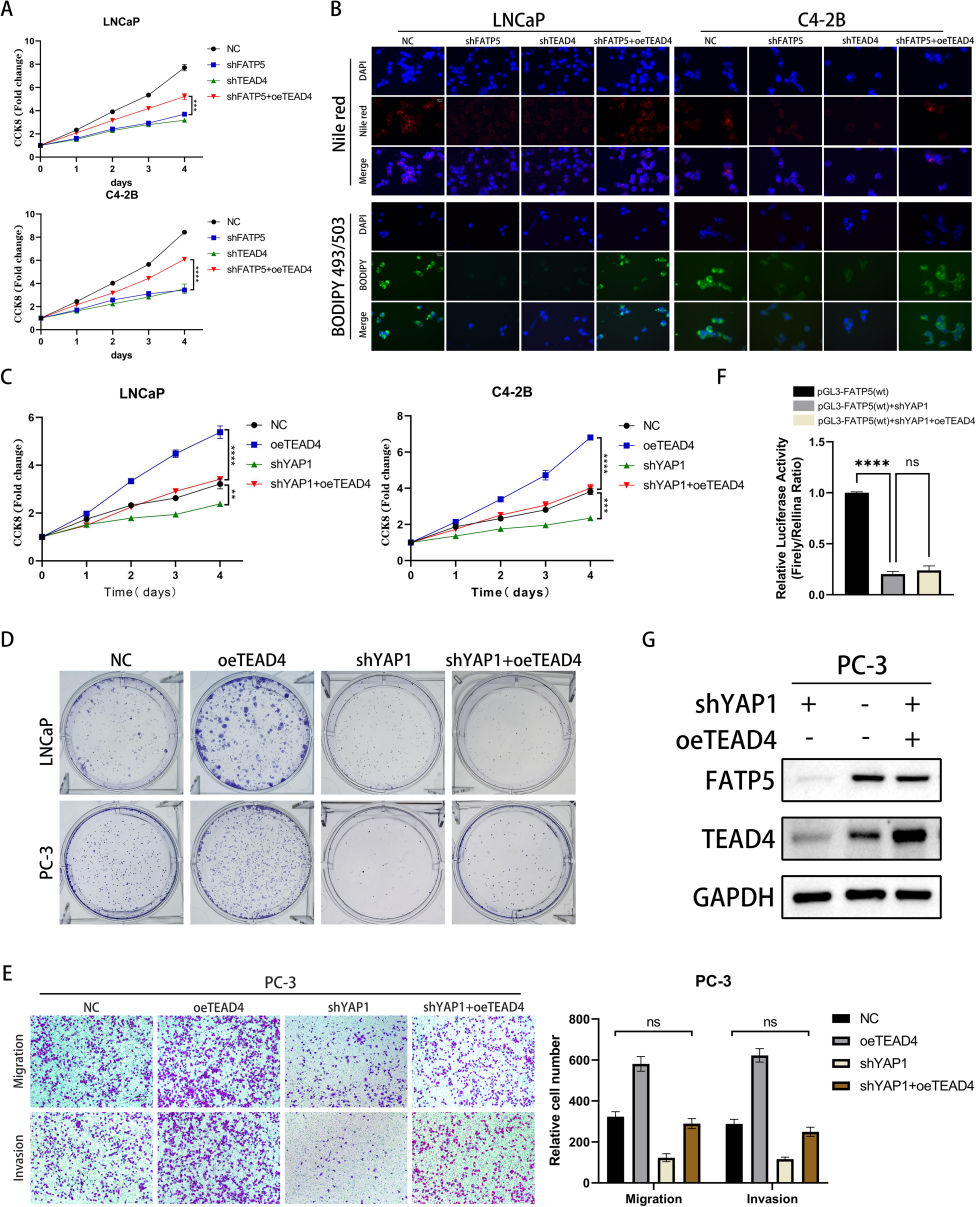
TEAD4 regulates the expression of FATP5 and the activity of PCa in a YAP1-dependent manner. **(A)** Conduct a CCK8 assay on the designated cells to assess the cell growth curve. **(B)** Present Nile red and BODIPY 493/503 staining images of the designated cells (Magnification: 400×). **(C)** Perform a CCK8 assay on the designated cells to determine the cell growth curve. **(D)** Perform colony formation assays on the designated cells and document the size and quantity of the resulting colonies. **(E)** Perform Transwell assays on the designated cells to assess their migration and invasion abilities (Magnification: 100×). Mean ± SD, n = 3. **(F)** Present the results of the dual-luciferase reporter gene assay conducted on the designated cells. **(G)** Evaluate the protein expression levels of FATP5 following the corresponding gene manipulation. **p < 0.01,***p < 0.001, and ****p < 0.0001. ns, no significance.

Subsequently, we performed *in vivo* validation of the impact of TEAD4 on proliferation. Upon TEAD4 knockdown, there was a significant decrease in tumor size, weight, and volume in mice (**Figures 5H–J**). To further establish the correlation between FATP5 and TEAD4 in PCa, we conducted functional rescue experiments. Utilizing the aforementioned methodology, we generated four cell line groups for experimentation: control cell line (NC), FATP5 knockdown cell line (shFATP5), TEAD4 knockdown cell line (shTEAD4), and FATP5 knockdown cell line with TEAD4 overexpression (shFATP5+oeTEAD4). As previously mentioned, knockdown of FATP5 significantly attenuated the migration and invasion capabilities of PCa cells. However, TEAD4 overexpression partially restored these abilities (**Figure 5K**). Proliferation capacity was assessed using the CCK8 assay, and consistent with the previous findings, TEAD4 overexpression partially reversed the growth inhibition caused by FATP5 knockdown (**Figure 6A**). Lipid staining results also demonstrated a partial recovery of lipid accumulation in PCa cells upon TEAD4 overexpression (**Figure 6B**). Hence, we can draw a consistent conclusion regarding the relationship between FATP5 and TEAD4, indicating that FATP5 primarily modulates the biological activity and lipid metabolism of PCa through the TEAD4-mediated Hippo signaling pathway.

### 3.6 TEAD4 regulates the expression of FATP5 and the activity of PCa in a YAP1-dependent manner

As a DNA anchor protein, TEAD4’s transcriptional reprogramming ability is largely dependent on its interaction with co-activators, such as YAP, which can be classified into two major classes: YAP-dependent and YAP-independent (21). To determine which type of regulation is involved in TEAD4-mediated regulation of FATP5, we established four cell line models: control cell line (NC), TEAD4 overexpression cell line (oeTEAD4), YAP1 knockdown cell line (shYAP1), and YAP1 knockdown cell line with TEAD4 overexpression (shYAP1+oeTEAD4). The results of CCK8 and clonogenic assays demonstrated that knockdown of YAP1 significantly attenuated the proliferative enhancement caused by TEAD4 overexpression (**Figures 6C, D**, **Supplementary Figure S3A**). Similarly, Transwell assays confirmed that the enhanced migration and proliferation induced by TEAD4 overexpression were reversed upon YAP1 knockdown (**Figure 6E**). Furthermore, we performed dual-luciferase reporter gene experiments to analyze the transcriptional regulation of FATP5 after YAP1 knockdown. The results showed that the transcriptional promotion of FATP5 by TEAD4 was greatly inhibited upon YAP1 knockout (**Figure 6F**). These findings were further supported by Western blot experiments (**Figure 6G**). Based on our previous experiments, the overexpression of FATP5 promotes YAP1 nuclear translocation, and nuclear YAP1, in turn, binds to TEAD4 to enhance the transcription of FATP5, forming a malignant cycle that promotes the progression of PCa. In summary, we can conclude that TEAD4 regulates the transcription of FATP5 in a YAP1-dependent manner, and the transcriptional activation of FATP5 promotes YAP1 nuclear localization, establishing a malignant cycle that enhances the biological functions of PCa.

### 3.7 FATP5 affects enzalutamide resistance and targeting the interaction of TEAD4 and YAP1 can reduce the activity of PCa

**Figure 7:**
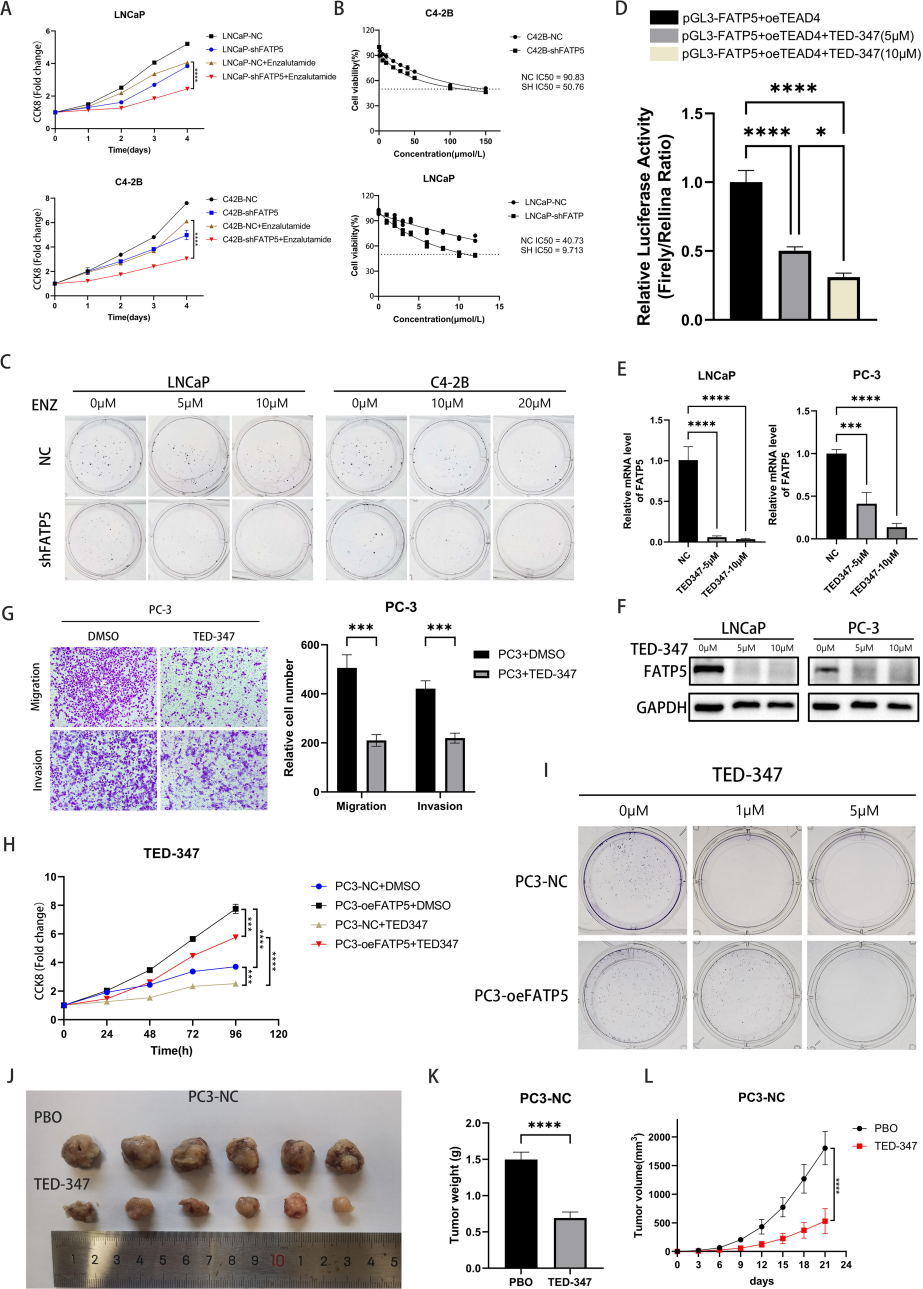
FATP5 affects enzalutamide resistance and targeting the interaction of TEAD4 and YAP1 can reduce the activity of PCa. **(A)** Conduct a CCK8 assay on the specified cells to determine the cell growth curve. **(B)** Determine the IC50 values for LNCaP and C4-2B cells. IC50:half maximal inhibitory concentration. **(C)** Perform colony formation assays after knocking down FATP5 and adding varying concentrations of Enzalutamide. **(D)** Measure the dual-luciferase activity in PC3 cells after treating them with different concentrations of TED-347. **(E, F)** Assess the mRNA and protein levels of FATP5 in PC-3 and LNCaP cells following treatment with different concentrations of TED-347. **(G)** Evaluate the migration and invasion capacities of cells after treatment with TED-347. **(H)** Conduct a CCK8 assay on the designated cells following the addition of TED-347 to determine the cell growth curve. **(I)** Conduct colony formation assays on the designated cells after treating them with varying concentrations of TED-347. **(J–L)** Implant tumors in mice and administer either a placebo or TED-347. After 21 days, record the tumor size, weight, and volume in the mice. Mean ± SD, n = 3. *p < 0.5, ***p < 0.001, and ****p < 0.0001.

Previous research has reported that the Hippo signaling pathway often plays a role in drug resistance, and in PCa, androgen deprivation therapy (ADT) frequently leads to drug resistance (22). Therefore, we hypothesized that the knockout of FATP5 may reverse PCa’s resistance to enzalutamide and enhance drug sensitivity. We conducted CCK8 experiments first and observed that knocking down FATP5 significantly increased the sensitivity to enzalutamide in both hormone-sensitive LNCaP cells and hormone-resistant C4-2B cells (**Figure 7A**). Clonogenic assays yielded consistent results, as the formation of colonies was more difficult in FATP5-knockdown cells compared to control cells when treated with varying concentrations of enzalutamide. Subsequently, we determined the IC50 values of the cell groups for enzalutamide and found a noticeable decrease in IC50 values after FATP5 knockdown, supporting our hypothesis. These findings collectively demonstrate that FATP5 can sensitize PCa cells to enzalutamide and partially reverse hormone resistance (**Figures 7B, C**, **Supplementary Figure S3B**).

TED-347 is a YAP1-selective inhibitor at the protein level, which dose- and time-dependently inhibits the binding of TEAD4 and YAP1 (23), subsequently affecting biological processes. We treated cells with different concentrations of TED-347 and assessed its impact on FATP5 transcription using dual-luciferase reporter gene experiments. The results indicated that higher TED-347 concentrations resulted in stronger inhibition of FATP5 transcription (**Figure 7D**). mRNA and protein level analyses also confirmed the inhibitory effect of TED-347 on FATP5 transcription (**Figures 7E, F**). Transwell assays revealed that TED-347 could influence the migration and invasion abilities of PCa cells (**Figure 7G**). Furthermore, we investigated the effect of TED-347 on PCa proliferation and demonstrated its ability to inhibit PCa cell growth. Interestingly, when conducting experiments in FATP5-overexpressing cell lines, we observed a decreased sensitivity to TED-347 (**Figures 7H, I**, **Supplementary Figure S3C**), suggesting a certain degree of resistance to TED-347 after FATP5 overexpression. Finally, we validated the inhibitory effect of TED-347 *in vivo*, and the results showed that administering TED-347 to mice significantly reduced tumor size, weight, and volume (**Figures 7J–L**). Furthermore, we repeated the experiment in PC3-oeFATP5 cell line, and the results demonstrated that TED-347 also inhibits the enhanced proliferation induced by FATP5 (**Supplementary Figure S4**).

## 4 Discussion

There is mounting evidence suggesting that the FATP family plays a significant role in various types of tumors. For instance, FATP1 has been shown to promote tumor progression in melanoma (8), while FATP3 contributes to immune suppression and tumor activity in lung cancer (24). FATP5 is overexpressed in colorectal cancer and can serve as a prognostic marker (25). These findings underscore the importance of the FATP family in tumorigenesis. However, the role of the FATP family in PCa remains unclear. FATP5 has been found to be upregulated in PCa tissues and cells, consistent with previous findings in colorectal cancer but contradictory to results observed in liver cancer (25, 26). These discoveries imply that FATP5 may exert its effects in multiple cancer types, and the tumor microenvironment and genetic background may influence the physiological function of FATP5 in different tumors. Through a series of *in vitro* and *in vivo* functional experiments, we have demonstrated that FATP5 may promote proliferation, migration, and invasion capabilities in PCa.

Our research has revealed that FATP5 is deeply involved in the lipid metabolism process of PCa. FATP5 is a human protein encoded by the SLC27A5 gene, with a molecular weight of approximately 70-80 kDa and consisting of 690 amino acids (27). It is typically expressed in tissues and cells, and plays a significant role in regulating the transport of exogenous fatty acids and maintaining intracellular lipid homeostasis. FATP5 likely mediates the entry of long-chain fatty acids (LCFAs) into cells by facilitating their translocation across the cell membrane. Furthermore, it catalyzes the ATP-dependent conversion of LCFA and VLCFA into acyl-CoA (28). Through a series of lipid staining experiments and lipidomics analysis, we have discovered that FATP5 enhances lipid accumulation in PCa and significantly increases the levels of various lipid metabolites. Additionally, we have validated the ability of FATP5 to enhance fatty acid uptake to some extent using fluorescence probes. These findings are consistent with previous studies on the function of FATP5 and demonstrate its important role in lipid metabolism in PCa. Moreover, the elevated lipid levels within PCa cells have been found to elevate cellular metabolism. We observed that overexpression of FATP5 leads to increased levels of ROS, NADP+/NADPH, and MDA within the cells, suggesting that the enhanced tumor biological functions resulting from FATP5 overexpression may be Partially attributed to its augmented lipid metabolism levels.

**Figure 8:**
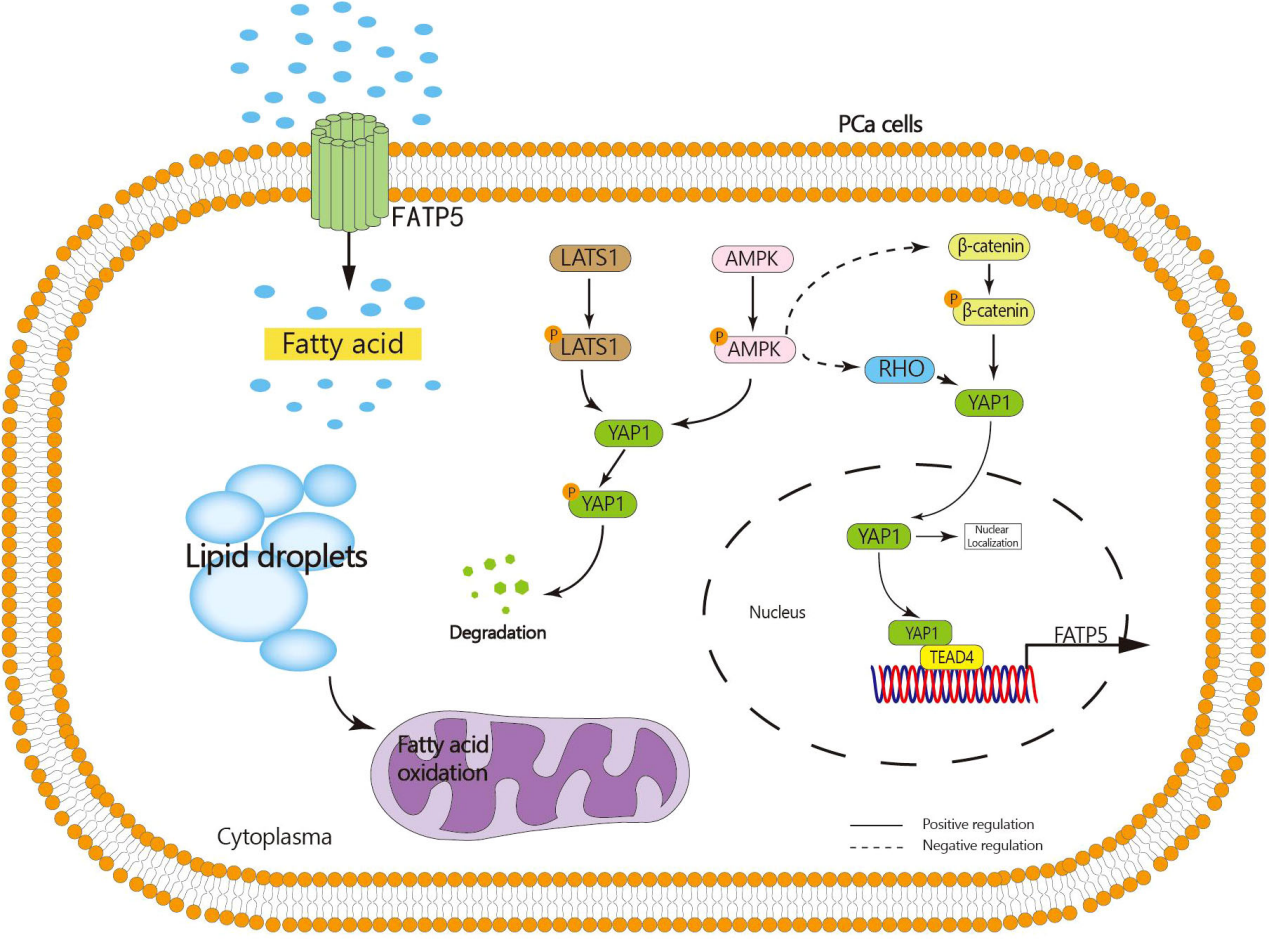
Graphical abstract: FATP5 promotes lipid metabolism in PCa cells, inhibits AMPK activation, facilitates nuclear translocation of YAP1, and nuclear YAP1 interacts with TEAD4 to enhance FATP5 transcription.

The results of whole transcriptome sequencing reveal a strong correlation between the transcription of FATP5 and the Hippo pathway. In recent years, the Hippo signaling pathway has been found to play a role in various biological processes of tumors, including proliferation, migration, and drug resistance (21). The key protein molecule in the Hippo pathway is YAP1. When the Hippo signaling pathway is inhibited, YAP1 undergoes decreased phosphorylation and degradation, leading to its translocation into the nucleus and subsequent promotion of downstream gene transcription (16). Our study demonstrates that knocking down FATP5 in PCa cells significantly reduces the nuclear levels of YAP1, indicating that FATP5 is capable of driving the nuclear translocation of YAP1. Further investigations revealed that the YAP1 nuclear translocation induced by FATP5 and activation of the Hippo signaling pathway are attributed to its inhibition of AMPK activation, which in turn promotes the activation of β-catenin and RHOA. Consequently, YAP1 nuclear translocation is facilitated. Additionally, the activation of AMPK itself promotes the cytoplasmic retention of YAP1, leading to its phosphorylation and subsequent degradation. Additionally, our research identifies TEAD4, a member of the TEAD transcription factor family, as being involved in the transcriptional process of FATP5, and this transcription is highly dependent on the interaction between TEAD4 and YAP1. Activation of FATP5 transcription occurs only when TEAD4 forms a transcriptional complex with YAP1. Therefore, nuclear YAP1 promotes the transcription of FATP5, and FATP5, in turn, enhances YAP1 nuclear localization, forming a malignant cycle that greatly facilitates the progression of PCa (**Figure 8**).

The Hippo signaling pathway has been reported to be implicated in drug resistance in tumors. Long-term androgen deprivation therapy (ADT) in PCa patients often leads to the development of irreversible castration-resistant PCa (CRPC) (29), highlighting the crucial need for novel therapeutic approaches. Our study reveals that knockdown of FATP5 enhances the sensitivity of PCa cells to enzalutamide, suggesting that FATP5 may serve as a potential therapeutic target for CRPC. Additionally, small molecule inhibitors targeting the interaction between TEAD4 and YAP1, such as TED-347, have already demonstrated excellent therapeutic efficacy in PCa cells.

However, our study also has certain limitations. For example, we did not investigate the specific mechanism by which FATP5 enhances sensitivity to enzalutamide. Additionally, it has been reported that the FATP family plays a crucial role in promoting the formation of an immunosuppressive tumor microenvironment. Therefore, our future research will focus on elucidating the precise mechanisms by which FATP5 mitigates enzalutamide resistance and its impact on the immune microenvironment. In summary, FATP5 exhibits upregulation in PCa, and TEAD4 mediates the transcription of FATP5 in a YAP1-dependent manner. Furthermore, FATP5 holds potential as a novel diagnostic biomarker and prognostic factor in PCa, offering new therapeutic options for the management of PCa.

In conclusion, the results of this study indicate that FATP5 plays a crucial regulatory role in the progression of PCa. Specifically, overexpression of FATP5 promotes both *in vivo* and *in vitro* progression of PCa and is deeply involved in PCa lipid metabolism, which is dependent on the interaction between TEAD4 and YAP1 in the Hippo signaling pathway. Therefore, targeting the expression of FATP5 or the interaction between TEAD4 and YAP1 may represent a promising therapeutic strategy to reduce PCa proliferation and inhibit disease progression.

## Data Availability

The raw data supporting the conclusions of this article will be made available by the authors, without undue reservation.The data presented in the study are deposited in the NCBI repository, accession number PRJNA1146434
