# Supplementary material for: FATP5 modulates biological activity and lipid metabolism in prostate cancer through the TEAD4-mediated Hippo signaling
Source: Front Oncol. 2024 Aug 19;14:1442911. doi: 10.3389/fonc.2024.1442911 (PMC11366587; doi:10.3389/fonc.2024.1442911)
Supplement: Supplementary file 1 [file DataSheet1.pdf]

1 Supplementary Figures and Tables

Supplementary Figure 1:

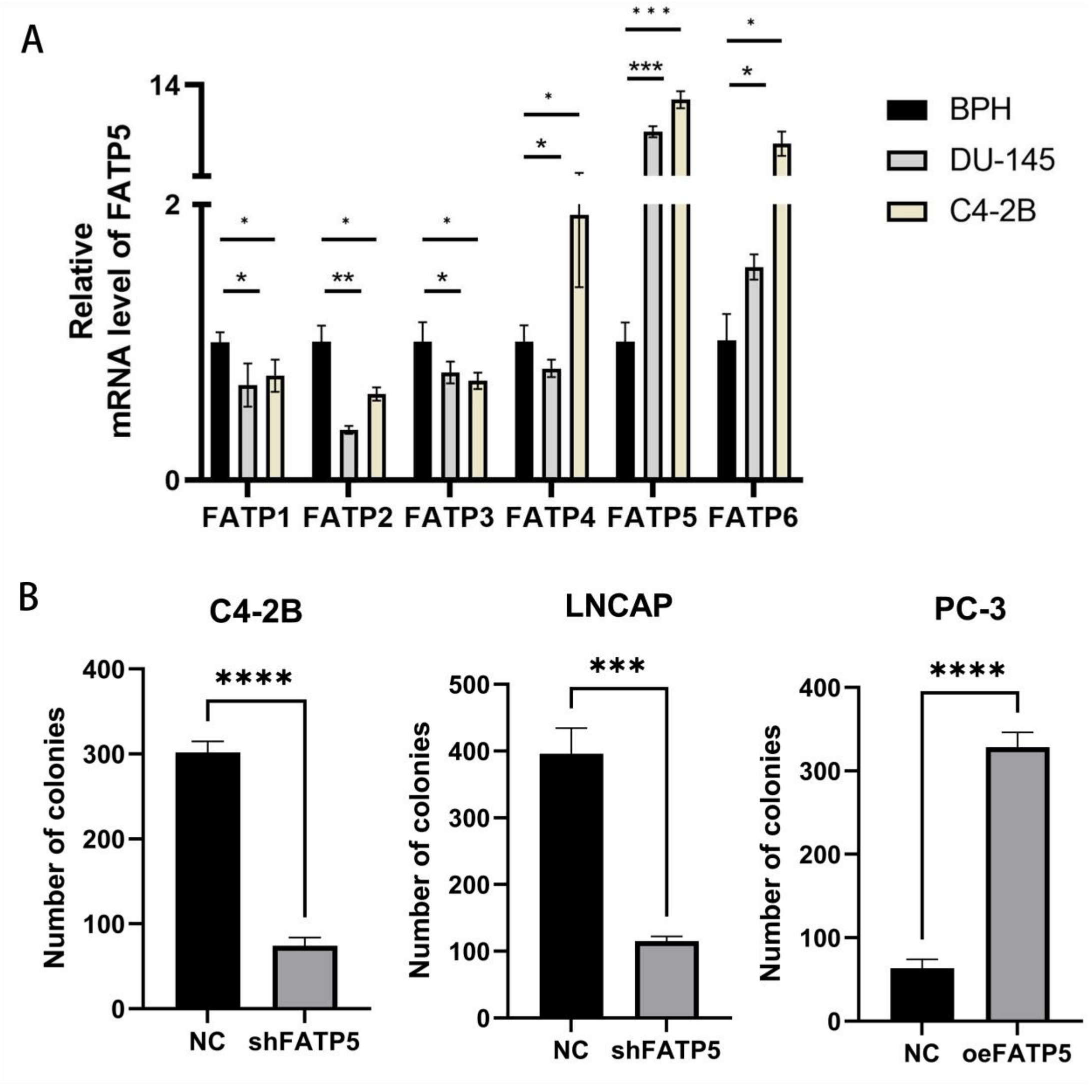

Figure S1. A The qPCR results revealed the mRNA expression levels of FATP5 in DU-145 and C4-2B cells. **B** The number of colonies after knocking down or overexpressing FATP5. *p < 0.5, **p < 0.01, ***p < 0.001, and ****p < 0.0001.

Supplementary Figure 2:

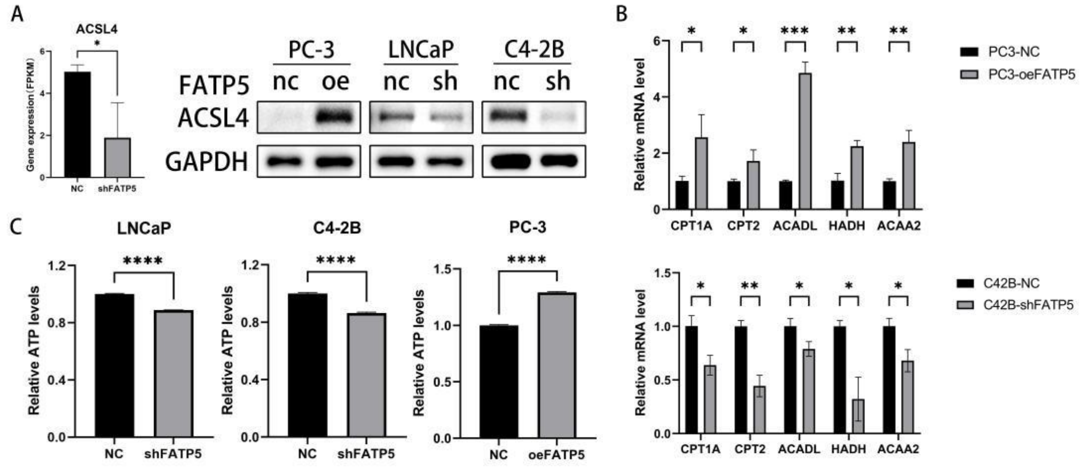

Figure S2.FATP5 enhances intracellular ATP levels by promoting fatty acid β-oxidation. (A) Specifies the expression of ACSL4 within cells. (B) Indicates the expression of key genes involved in β-oxidation. (C) Specifies the intracellular ATP levels within cells.*p < 0.5, **p < 0.01, ***p < 0.001, and ****p < 0.0001.

Supplementary Figure 3:

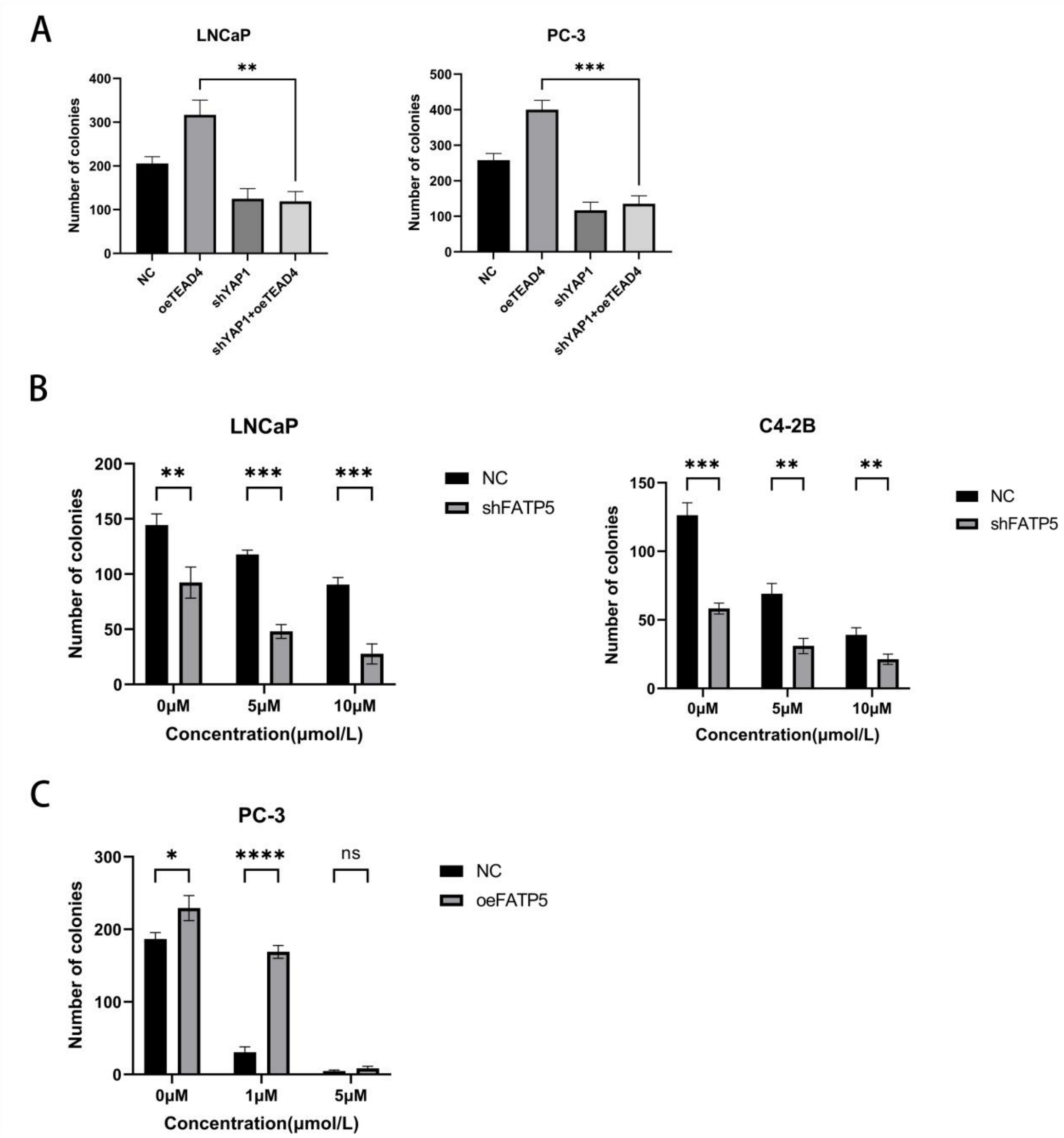

Figure S3. A The number of colonies in different treated cells. **B** The number of colonies at different concentrations of enzalutamide. **C** The number of colonies at different concentrations of TED-347. *p < 0.5, **p < 0.01, ***p < 0.001, and ****p < 0.0001.

Supplementary Figure 4:

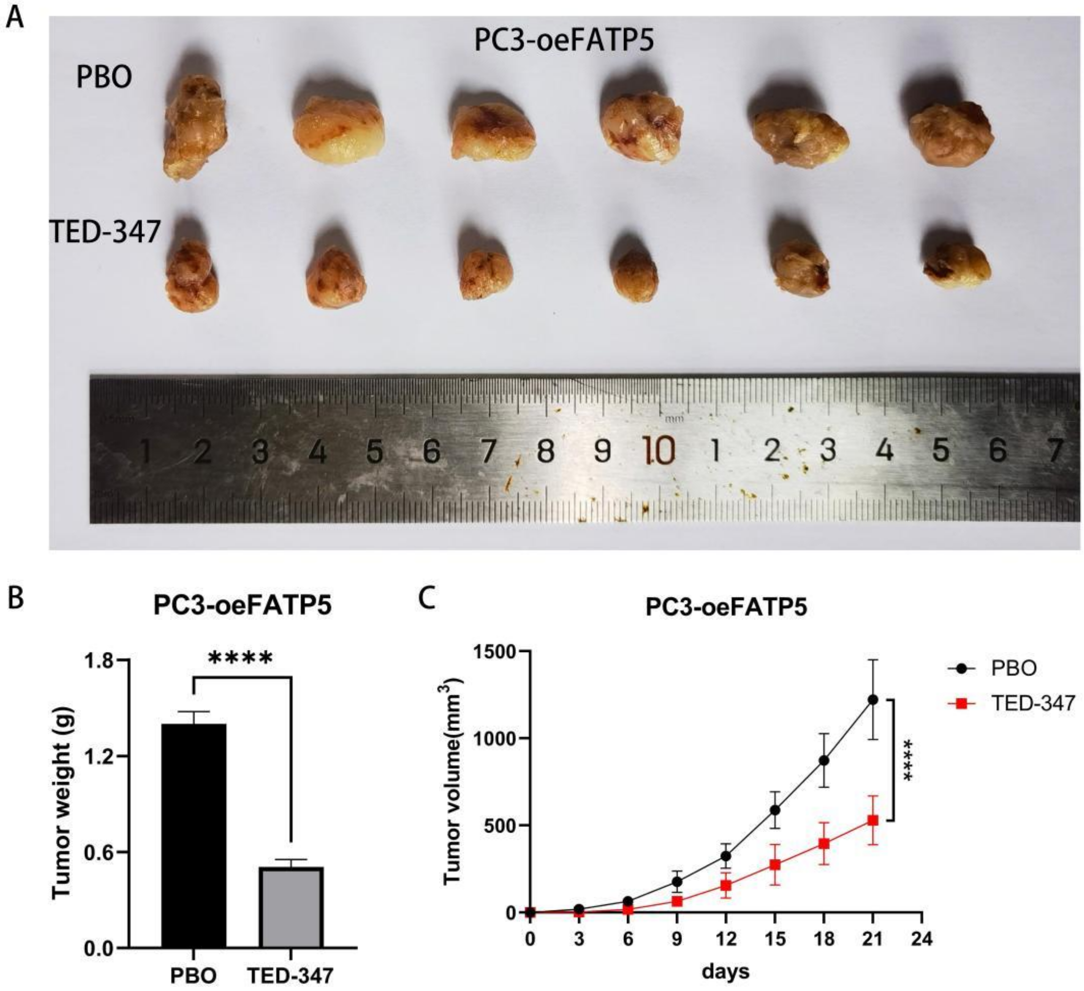

Figure S4.TED-347 significantly inhibits the enhanced proliferation induced by FATP5 in vivo. (A) Tumor size. (B) Tumor weight. (C) Tumor volume.*p < 0.5, **p < 0.01, ***p < 0.001, and ****p < 0.0001.

Supplementary Figure 5:

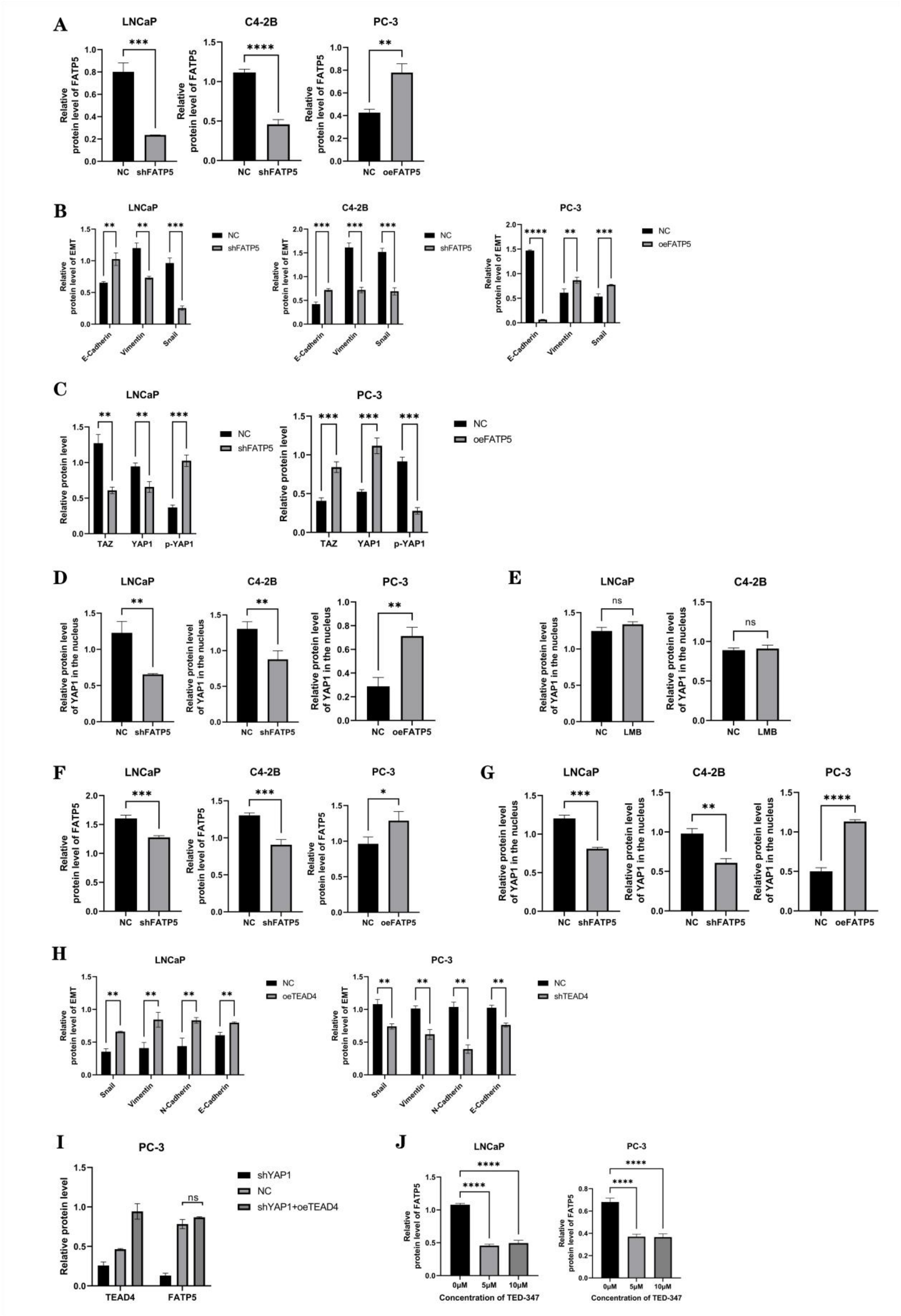

Figure S5. A The quantitative results of Western blot analysis for Figure 2B. **B** The quantitative results of Western blot analysis for Figure 2G. **C** The quantitative results of Western blot analysis for Figure 4F. **D** The quantitative results of Western blot analysis for Figure 4G. **E** The quantitative results of Western blot analysis for Figure 4H. **F** The quantitative results of Western blot analysis for Figure 5C. **G** The quantitative results of Western blot analysis for Figure 5D. **H** The quantitative results of Western blot analysis for Figure 5E. **I** The quantitative results of Western blot analysis for Figure 6G. **J** The quantitative results of Western blot analysis for Figure 7F.

1.2 Supplementary Table

Supplementary Table 1:

Table 1: The primers used and shRNA		
**qPCR primers**		
GAPDH	forward	GGAGCGAGATCCCTCCAAAAT
	reverse	GGCTGTTGTCATACTTCTCATGG
ACATB	forward	CATGTACGTTGCTATCCAGGC
	reverse	CTCCTTAATGTCACGCACGAT
FATP1	forward	GGGGCAGTGTCTCATCTATGG
	reverse	CCGATGTACTGAACCACCGT
FATP2	forward	TTTCCGCCATCTACACAGTCC
	reverse	CGTAGGTGAGAGTCTCGTCG
FATP3	forward	GAGAGCCAATTCGGGACCC
	reverse	GCCGGAAGACATCCTTTAGCAA
FATP4	forward	GTGAAGGCAAAGGTGCGAC
	reverse	CGGAAGGTCCAGTGGGTATC
FATP5	forward	CATGGCGTGACAGTGATCCT
	reverse	CAGCCCGTAGTCCATTGCC
FATP6	forward	CTTCTGTCATGGCTAACAGTTCT
	reverse	AGGTTTCCGAGGTTGTCTTTTG
TEAD4	forward	GAACGGGGACCCTCCAATG
	reverse	GCGAGCATACTCTGTCTCAAC
CPT1	forward	TCCAGTTGGCTTATCGTGGTG
	reverse	TCCAGAGTCCGATTGATTTTTGC
CPT2	forward	CATACAAGCTACATTTCGGGACC
	reverse	AGCCCGGAGTGTCTTCAGAA
ACADL	forward	AGGGGATCTGTACTCCGCAG
	reverse	CTCTGTCATTGCTATTGCACCA
HADH	forward	ACCAGGCAGTTCATGCGTT
	reverse	ACGTGCTTGACGATTATCTTCTT
ACAA2	forward	CTGCTCCGAGGTGTGTTTGTA
	reverse	GGCAGCAAATTCAGACAAGTCA
**shRNA sequence**		
shYAP1		GCCACCAAGCTAGATAAAGAA
shTEAD4		GAGACAGAGTATGCTCGCTAT
shFATP5		ACATGGGCTTAGTCAACTATG
**Molecular cloning primers**		
Pro-FATP5	forward	GTGCTAGCCCGGGCTCGACCCCTCCCCTAACCCAGAATGA
	reverse	TTAGATCGCAGATCTCGACCAGCCCAAGTAGCACGCAA
oeTEAD4	forward	GACGATGACAAGCTCGAG
	reverse	GTCCTTGTAATCCTCGAG

Table S1:The primers and shRNA sequence.

Supplementary Table 2:

location	number	age	gender	Organs	pathological diagnosis	TNM	Grade	Stage	Type	Pathological ID	Gleason Score	Gleason Grade
A1	1	69	male	prostate	adenocarcinoma	T2N0M0	–	IIIC	Cancer	A1837950	5+4=9	5
A2	2	69	male	prostate	Normal prostate gland adjacent to cancer	–	–	–	NAT	A1837950	–	–
A3	3	72	male	prostate	adenocarcinoma	T3bN0M0	–	IIIB	Cancer	A1841661	4+3=7	3
A4	4	72	male	prostate	Normal prostate gland	–	–	–	NAT	A1841661	–	–
A5	5	78	male	prostate	adenocarcinoma	T3bN0M0	–	IIIC	Cancer	A1844554	5+4=9	5
A6	6	78	male	prostate	Normal prostate gland	–	–	–	NAT	A1844554	–	–
A7	7	71	male	prostate	adenocarcinoma	T2N0M0	–	IIC	Cancer	A1846805	3+5=8	4
A8	8	71	male	prostate	Normal prostate gland	–	–	–	NAT	A1846805	–	–
A9	9	81	male	prostate	adenocarcinoma	T3bN0M0	–	IIIC	Cancer	A1920399	5+5=10	5
A10	10	81	male	prostate	Normal prostate gland	–	–	–	NAT	A1920399	–	–
A11	11	79	male	prostate	adenocarcinoma	T3bN0M0	–	IIIB	Cancer	A1852718	4+3=7	3
A12	12	79	male	prostate	Normal prostate gland	–	–	–	NAT	A1852718	–	–
A13	13	50	male	prostate	adenocarcinoma	T2N0M0	–	IIIC	Cancer	A1855208	4+5=9	5
A14	14	50	male	prostate	Normal prostate gland	–	–	–	NAT	A1855208	–	–
A15	15	54	male	prostate	adenocarcinoma	T4N0M0	–	IIIB	Cancer	A2167832	3+4=7	2
A16	16	54	male	prostate	Normal prostate gland	–	–	–	NAT	A2167832	–	–
A17	17	69	male	prostate	adenocarcinoma	T2N0M0	–	IIC	Cancer	A2155423	3+4=7	3
A18	18	69	male	prostate	Normal prostate gland	–	–	–	NAT	A2155423	–	–
A19	19	58	male	prostate	adenocarcinoma	T3bN0M0	–	IIIC	Cancer	A2158836	5+4=9	5
A20	20	58	male	prostate	Normal prostate gland	–	–	–	NAT	A2158836	–	–
B1	21	74	male	prostate	adenocarcinoma	T3bN0M0	–	IIIC	Cancer	A1855320	5+4=9	5
B2	22	74	male	prostate	Normal prostate gland	–	–	–	NAT	A1855320	–	–
B3	23	76	male	prostate	adenocarcinoma	T3bN0M0	–	IIIC	Cancer	A1855415	4+5=9	5
B4	24	76	male	prostate	Normal prostate gland	–	–	–	NAT	A1855415	–	–
B5	25	72	male	prostate	adenocarcinoma	T3bN0M0	–	IIIB	Cancer	A2059747	4+3=7	3
B6	26	72	male	prostate	Normal prostate gland	–	–	–	NAT	A2059747	–	–
B7	27	68	male	prostate	adenocarcinoma	T2N0M0	–	IIB	Cancer	A1938773	3+4=7	2
B8	28	68	male	prostate	Normal prostate gland	–	–	–	NAT	A1938773	–	–
B9	29	67	male	prostate	adenocarcinoma	T4N0M0	–	IIIC	Cancer	A1939230	4+5=9	5
B10	30	67	male	prostate	Normal prostate gland	–	–	–	NAT	A1939230	–	–
B11	31	75	male	prostate	adenocarcinoma	T3bN0M0	–	IIIC	Cancer	A1939577	5+4=9	5
B12	32	75	male	prostate	Normal prostate gland	–	–	–	NAT	A1939577	–	–
B13	33	73	male	prostate	adenocarcinoma	T2N0M0	–	IIIC	Cancer	A1941570	4+5=9	5
B14	34	73	male	prostate	Normal prostate gland	–	–	–	NAT	A1941570	–	–
B15	35	70	male	prostate	adenocarcinoma	T3bN0M0	–	IIIC	Cancer	A2160110	5+5=10	5
B16	36	70	male	prostate	Normal prostate gland	–	–	–	NAT	A2160110	–	–
B17	37	69	male	prostate	adenocarcinoma	T3bN0M0	–	IIIB	Cancer	A2138951	4+4=8	4
B18	38	69	male	prostate	Normal prostate gland	–	–	–	NAT	A2138951	–	–
B19	39	68	male	prostate	adenocarcinoma	T3bN0M0	–	IIIC	Cancer	A2161145	4+5=9	5
B20	40	68	male	prostate	Normal prostate gland	–	–	–	NAT	A2161145	–	–
C1	41	67	male	prostate	adenocarcinoma	T3bN0M0	–	IIIC	Cancer	A1941943	4+5=9	5
C2	42	67	male	prostate	Normal prostate gland	–	–	–	NAT	A1941943	–	–
C3	43	68	male	prostate	adenocarcinoma	T3bN0M0	–	IIIC	Cancer	A2058713	4+5=9	5
C4	44	68	male	prostate	Normal prostate gland	–	–	–	NAT	A2058713	–	–
C5	45	64	male	prostate	adenocarcinoma	T2N0M0	–	IIIC	Cancer	A1947673	4+5=9	5
C6	46	64	male	prostate	Normal prostate gland	–	–	–	NAT	A1947673	–	–
C7	47	70	male	prostate	adenocarcinoma	T3bN0M0	–	IIIC	Cancer	A2150163	5+4=9	5
C8	48	70	male	prostate	Normal prostate gland	–	–	–	NAT	A2150163	–	–
C9	49	64	male	prostate	adenocarcinoma	T4N0M0	–	IIIC	Cancer	A1953479	4+5=9	5
C10	50	64	male	prostate	Normal prostate gland	–	–	–	NAT	A1953479	–	–

C11	51	75	male	prostate	adenocarcinoma	T3bN0M0	–	IIIB	Cancer	A2151466	4+3=7	3
C12	52	75	male	prostate	Normal prostate gland	–	–	–	NAT	A2151466	–	–
C13	53	66	male	prostate	adenocarcinoma	T2N0M0	–	IIIC	Cancer	A2151142	4+5=9	5
C14	54	66	male	prostate	Normal prostate gland	–	–	–	NAT	A2151142	–	–
C15	55	71	male	prostate	adenocarcinoma	T3bN0M0	–	IIIC	Cancer	A2129367	5+4=9	5
C16	56	71	male	prostate	Normal prostate gland	–	–	–	NAT	A2129367	–	–
C17	57	75	male	prostate	adenocarcinoma	T2N0M0	–	IIIC	Cancer	A2141044	5+4=9	5
C18	58	75	male	prostate	Normal prostate gland	–	–	–	NAT	A2141044	–	–
C19	59	63	male	prostate	adenocarcinoma	T2N0M0	–	I	Cancer	A2141731	3+3=6	1
C20	60	63	male	prostate	Normal prostate gland	–	–	–	NAT	A2141731	–	–
D1	61	64	male	prostate	adenocarcinoma	T4N0M0	–	IIIC	Cancer	A1821269	5+4=9	5
D2	62	64	male	prostate	Normal prostate gland	–	–	–	NAT	A1821269	–	–
D3	63	77	male	prostate	adenocarcinoma	T2N0M0	–	IIIA	Cancer	A1829242	4+4=8	4
D4	64	77	male	prostate	Normal prostate gland	–	–	–	NAT	A1829242	–	–
D5	65	69	male	prostate	adenocarcinoma	T3N0M0	–	IIIC	Cancer	A2153980	4+5=9	5
D6	66	69	male	prostate	Normal prostate gland	–	–	–	NAT	A2153980	–	–
D7	67	66	male	prostate	adenocarcinoma	T3bN0M0	–	IIIC	Cancer	A2151651	4+5=9	5
D8	68	66	male	prostate	Normal prostate gland	–	–	–	NAT	A2151651	–	–
D9	69	78	male	prostate	adenocarcinoma	T3bN0M0	–	IIIB	Cancer	A1954023	4+3=7	3
D10	70	78	male	prostate	Normal prostate gland	–	–	–	NAT	A1954023	–	–
D11	71	72	male	prostate	adenocarcinoma	T2bN0M0	–	IIB	Cancer	A1958383	3+4=7	2
D12	72	72	male	prostate	Normal prostate gland	–	–	–	NAT	A1958383	–	–
D13	73	76	male	prostate	adenocarcinoma	T3bN0M0	–	IIIC	Cancer	A2123839	4+5=9	5
D14	74	76	male	prostate	Normal prostate gland	–	–	–	NAT	A2123839	–	–
D15	75	66	male	prostate	adenocarcinoma	T3bN0M0	–	IIIB	Cancer	A2140688	4+4=8	4
D16	76	66	male	prostate	Normal prostate gland	–	–	–	NAT	A2140688	–	–
D17	77	70	male	prostate	adenocarcinoma	T2N0M0	–	IIB	Cancer	A2139990	3+4=7	2
D18	78	70	male	prostate	Normal prostate gland	–	–	–	NAT	A2139990	–	–
D19	79	71	male	prostate	adenocarcinoma	T3bN0M0	–	IIIC	Cancer	A2129366	5+4=9	5
D20	80	71	male	prostate	Normal prostate gland	–	–	–	NAT	A2129366	–	–
E1	81	80	male	prostate	adenocarcinoma	T3bN0M0	–	IIIC	Cancer	A1959879	4+5=9	5
E2	82	80	male	prostate	Normal prostate gland	–	–	–	NAT	A1959879	–	–
E3	83	83	male	prostate	adenocarcinoma	T3bN0M0	–	IIIB	Cancer	A1962742	3+4=7	2
E4	84	83	male	prostate	Normal prostate gland	–	–	–	NAT	A1962742	–	–
E5	85	62	male	prostate	adenocarcinoma	T2N0M0	–	IIC	Cancer	A1965777	4+4=8	4
E6	86	62	male	prostate	Normal prostate gland	–	–	–	NAT	A1965777	–	–
E7	87	75	male	prostate	adenocarcinoma	T2aN0M0	–	IIIC	Cancer	A1965800	4+5=9	5
E8	88	75	male	prostate	Normal prostate gland	–	–	–	NAT	A1965800	–	–
E9	89	65	male	prostate	adenocarcinoma	T2aN0M0	–	IIB	Cancer	A1966807	3+4=7	2
E10	90	65	male	prostate	Normal prostate gland	–	–	–	NAT	A1966807	–	–
E11	91	68	male	prostate	adenocarcinoma	T3bN0M0	–	IIIC	Cancer	A2030103	4+5=9	5
E12	92	68	male	prostate	Normal prostate gland	–	–	–	NAT	A2030103	–	–
E13	93	62	male	prostate	adenocarcinoma	T2N0M0	–	IIC	Cancer	A2032136	4+4=8	4
E14	94	62	male	prostate	Normal prostate gland	–	–	–	NAT	A2032136	–	–
E15	95	79	male	prostate	adenocarcinoma	T2N0M0	–	IIIC	Cancer	A2167651	4+5=9	5
E16	96	79	male	prostate	Normal prostate gland	–	–	–	NAT	A2167651	–	–
E17	97	58	male	prostate	adenocarcinoma	T2N0M0	–	IIIC	Cancer	A2157041	5+4=9	5
E18	98	58	male	prostate	Normal prostate gland	–	–	–	NAT	A2157041	–	–
E19	99	65	male	prostate	adenocarcinoma	T2N0M0	–	IIC	Cancer	A1965766	4+4=8	4
E20	100	65	male	prostate	Normal prostate gland	–	–	–	NAT	A1965766	–	–
F1	101	78	male	prostate	adenocarcinoma	T3bN0M0	–	IIIC	Cancer	A2033524	4+5=9	5
F2	102	78	male	prostate	Normal prostate gland	–	–	–	NAT	A2033524	–	–
F3	103	63	male	prostate	adenocarcinoma	T2N0M0	–	IIIC	Cancer	A2038328	4+5=9	5
F4	104	63	male	prostate	Normal prostate gland	–	–	–	NAT	A2038328	–	–
F5	105	67	male	prostate	adenocarcinoma	T2N0M0	–	IIB	Cancer	A2039685	3+4=7	2
F6	106	67	male	prostate	Normal prostate gland	–	–	–	NAT	A2039685	–	–
F7	107	67	male	prostate	adenocarcinoma	T4N0M0	–	IIIB	Cancer	A2042095	4+4=8	4
F8	108	67	male	prostate	Normal prostate gland	–	–	–	NAT	A2042095	–	–
F9	109	57	male	prostate	adenocarcinoma	T3bN0M0	–	IIIB	Cancer	A2045054	3+4=7	2
F10	110	57	male	prostate	Normal prostate gland	–	–	–	NAT	A2045054	–	–

F11	111	68	male	prostate	adenocarcinoma	T3bN1M0	–	IVA	Cancer	A2045867	4+4=8	4
F12	112	68	male	prostate	Normal prostate gland	–	–	–	NAT	A2045867	–	–
F13	113	83	male	prostate	adenocarcinoma	T3bN0M0	–	IIIC	Cancer	A2046414	5+4=9	5
F14	114	83	male	prostate	Normal prostate gland	–	–	–	NAT	A2046414	–	–
F15	115	76	male	prostate	adenocarcinoma	T3bN0M0	–	IIIB	Cancer	A2152477	4+3=7	3
F16	116	76	male	prostate	Normal prostate gland	–	–	–	NAT	A2152477	–	–
F17	117	74	male	prostate	adenocarcinoma	T2N0M0	–	IIB	Cancer	A2018175	3+4=7	2
F18	118	74	male	prostate	Normal prostate gland	–	–	–	NAT	A2018175	–	–
F19	119	72	male	prostate	adenocarcinoma	T2N0M0	–	IIC	Cancer	A1837949	4+4=8	4
F20	120	72	male	prostate	Normal prostate gland	–	–	–	NAT	A1837949	–	–
G1	121	72	male	prostate	adenocarcinoma	T3bN0M0	–	IIIC	Cancer	A2054514	5+4=9	5
G2	122	72	male	prostate	Normal prostate gland	–	–	–	NAT	A2054514	–	–
G3	123	64	male	prostate	adenocarcinoma	T2N0M0	–	IIIC	Cancer	A2055579	4+5=9	5
G4	124	64	male	prostate	Normal prostate gland	–	–	–	NAT	A2055579	–	–
G5	125	77	male	prostate	adenocarcinoma	T3bN0M0	–	IIIC	Cancer	A2056945	4+5=9	5
G6	126	77	male	prostate	Normal prostate gland	–	–	–	NAT	A2056945	–	–
G7	127	79	male	prostate	adenocarcinoma	–	–	–	Cancer	A2057937	–	–
G8	128	79	male	prostate	Normal prostate gland	T3bN0M0	–	IIIB	NAT	A2057937	4+4=8	4
G9	129	68	male	prostate	adenocarcinoma	T4N1M0	–	IVA	Cancer	A2016320	4+5=9	5
G10	130	68	male	prostate	Normal prostate gland	–	–	–	NAT	A2016320	–	–
G11	131	74	male	prostate	adenocarcinoma	T2N0M0	–	IIB	Cancer	A2018177	3+4=7	2
G12	132	74	male	prostate	Normal prostate gland	–	–	–	NAT	A2018177	–	–
G13	133	65	male	prostate	adenocarcinoma	T3bN0M0	–	IIIB	Cancer	A2020871	3+5=8	4
G14	134	65	male	prostate	Normal prostate gland	–	–	–	NAT	A2020871	–	–
G15	135	72	male	prostate	adenocarcinoma	T3bN0M0	–	IIIB	Cancer	A2059744	4+3=7	3
G16	136	72	male	prostate	Normal prostate gland	–	–	–	NAT	A2059744	–	–
G17	137	66	male	prostate	adenocarcinoma	T2N0M0	–	IIIC	Cancer	A2151139	4+5=9	5
G18	138	66	male	prostate	Normal prostate gland	–	–	–	NAT	A2151139	–	–
G19	139	64	male	prostate	adenocarcinoma	T2N0M0	–	IIB	Cancer	A2114604	3+4=7	2
G20	140	64	male	prostate	Normal prostate gland	–	–	–	NAT	A2114604	–	–
H1	141	73	male	prostate	adenocarcinoma	T3bN0M0	–	IIIC	Cancer	A2050534	4+5=9	5
H2	142	73	male	prostate	Normal prostate gland	–	–	–	NAT	A2050534	–	–
H3	143	75	male	prostate	adenocarcinoma	T3bN0M0	–	IIIC	Cancer	A2062795	5+5=10	5
H4	144	75	male	prostate	Normal prostate gland	–	–	–	NAT	A2062795	–	–
H5	145	67	male	prostate	adenocarcinoma	T3bN0M0	–	IIIC	Cancer	A2116984	5+4=9	5
H6	146	67	male	prostate	Normal prostate gland	–	–	–	NAT	A2116984	–	–
H7	147	66	male	prostate	adenocarcinoma	T3bN0M0	–	IIIC	Cancer	A2122788	5+5=10	5
H8	148	66	male	prostate	Normal prostate gland	–	–	–	NAT	A2122788	–	–
H9	149	64	male	prostate	adenocarcinoma	T2N0M0	–	IIB	Cancer	A2050727	3+4=7	2
H10	150	64	male	prostate	Normal prostate gland	–	–	–	NAT	A2050727	–	–
H11	151	76	male	prostate	adenocarcinoma	T3bN0M0	–	IIIB	Cancer	A2052477	4+3=7	3
H12	152	76	male	prostate	Normal prostate gland	–	–	–	NAT	A2052477	–	–
H13	153	70	male	prostate	adenocarcinoma	T2N0M0	–	IIB	Cancer	A2056884	3+4=7	2
H14	154	70	male	prostate	Normal prostate gland	–	–	–	NAT	A2056884	–	–
H15	155	64	male	prostate	adenocarcinoma	T2N0M0	–	IIC	Cancer	A2032138	4+4=8	4
H16	156	64	male	prostate	Normal prostate gland	–	–	–	NAT	A2032138	–	–
H17	157	77	male	prostate	adenocarcinoma	T3bN0M0	–	IIIC	Cancer	A1855318	5+4=9	5
H18	158	77	male	prostate	Normal prostate gland	–	–	–	NAT	A1855318	–	–
H19	159	78	male	prostate	adenocarcinoma	T3bN0M0	–	IIIC	Cancer	A1855400	4+5=9	5
H20	160	78	male	prostate	Normal prostate gland	–	–	–	NAT	A1855400	–	–
I1	161	60	male	prostate	adenocarcinoma	T2N0M0	–	IIB	Cancer	A2146055	3+4=7	2
I2	162	60	male	prostate	Normal prostate gland	–	–	–	NAT	A2146055	–	–
I3	163	69	male	prostate	adenocarcinoma	T2N0M0	–	IIIC	Cancer	A2146838	5+5=10	5
I4	164	69	male	prostate	Normal prostate gland	–	–	–	NAT	A2146838	–	–
I5	165	75	male	prostate	adenocarcinoma	T3bN1M0	–	IVA	Cancer	A2147486	5+4=9	5
I6	166	75	male	prostate	Normal prostate gland	–	–	–	NAT	A2147486	–	–
I7	167	68	male	prostate	adenocarcinoma	T3bN0M0	–	IIIC	Cancer	A2165406	4+5=9	5
I8	168	68	male	prostate	Normal prostate gland	–	–	–	NAT	A2165406	–	–
I9	169	65	male	prostate	adenocarcinoma	T3bN0M0	–	IIIC	Cancer	A2161885	4+5=9	5
I10	170	65	male	prostate	Normal prostate gland	–	–	–	NAT	A2161885	–	–

I11	171	76	male	prostate	adenocarcinoma	T3bN0M0	–	IIIB	Cancer	A2162100	3+5=8	4
I12	172	76	male	prostate	Normal prostate gland	–	–	–	NAT	A2162100	–	–
I13	173	76	male	prostate	adenocarcinoma	T2N0M0	–	IIB	Cancer	A2163691	3+4=7	2
I14	174	76	male	prostate	Normal prostate gland	–	–	–	NAT	A2163691	–	–
I15	175	66	male	prostate	adenocarcinoma	T2N0M0	–	IIIC	Cancer	A2151138	4+5=9	5
I16	176	66	male	prostate	Normal prostate gland	–	–	–	NAT	A2151138	–	–
I17	177	79	male	prostate	adenocarcinoma	T2N0M0	–	IIC	Cancer	A2154541	3+4=7	3
I18	178	79	male	prostate	Normal prostate gland	–	–	–	NAT	A2154541	–	–
I19	179	76	male	prostate	adenocarcinoma	T2N0M0	–	IIB	Cancer	A2018174	3+4=7	2
I20	180	76	male	prostate	Normal prostate gland	–	–	–	NAT	A2018174	–	–
J1	181	75	male	prostate	adenocarcinoma	T1aN0M0	–	I	Cancer	A2130358	3+3=6	1
J2	182	75	male	prostate	Normal prostate gland	–	–	–	NAT	A2130358	–	–
J3	183	79	male	prostate	adenocarcinoma	T2N0M0	–	IIIC	Cancer	A2167650	4+5=9	5
J4	184	79	male	prostate	Normal prostate gland	–	–	–	NAT	A2167650	–	–
J5	185	62	male	prostate	adenocarcinoma	T2N0M0	–	I	Cancer	A2170872	3+3=6	1
J6	186	62	male	prostate	Normal prostate gland	–	–	–	NAT	A2170872	–	–
J7	187	85	male	prostate	adenocarcinoma	T2N0M0	–	IIB	Cancer	A2167548	3+4=7	2
J8	188	85	male	prostate	Normal prostate gland	–	–	–	NAT	A2167548	–	–
J9	189	76	male	prostate	adenocarcinoma	T2N0M0	–	IIIC	Cancer	A2166677	4+5=9	5
J10	190	76	male	prostate	Normal prostate gland	–	–	–	NAT	A2166677	–	–
J11	191	69	male	prostate	adenocarcinoma	T2N0M0	–	IIC	Cancer	A2170306	4+3=7	3
J12	192	69	male	prostate	Normal prostate gland	–	–	–	NAT	A2170306	–	–
J13	193	68	male	prostate	adenocarcinoma	T3bN0M0	–	IIIB	Cancer	A2167912	4+3=7	3
J14	194	68	male	prostate	Normal prostate gland	–	–	–	NAT	A2167912	–	–
J15	195	64	male	prostate	adenocarcinoma	T2N0M0	–	IIA	Cancer	A2135021	3+3=6	1
J16	196	64	male	prostate	Normal prostate gland	–	–	–	NAT	A2135021	–	–
J17	197	74	male	prostate	adenocarcinoma	T3bN0M0	–	IIIB	Cancer	A2059743	4+3=7	3
J18	198	74	male	prostate	Normal prostate gland	–	–	–	NAT	A2059743	–	–
J19	199	68	male	prostate	adenocarcinoma	T4N0M0	–	IIIC	Cancer	A1939224	4+5=9	5

Table S2: Clinical information about the patient in the Tissue Microarray
